# Safety and vaccine-induced HIV-1 immune responses in healthy volunteers following a late MVA-B boost 4 years after the last immunization

**DOI:** 10.1371/journal.pone.0186602

**Published:** 2017-10-24

**Authors:** Alberto C. Guardo, Carmen Elena Gómez, Vicens Díaz-Brito, Judit Pich, Joan Albert Arnaiz, Beatriz Perdiguero, Juan García-Arriaza, Nuria González, Carlos O. S. Sorzano, Laura Jiménez, José Luis Jiménez, María Ángeles Muñoz-Fernández, José M Gatell, José Alcamí, Mariano Esteban, Juan Carlos López Bernaldo de Quirós, Felipe García, Montserrat Plana, for the RISVAC02boost study

**Affiliations:** 1 Immunopathology and Cellular Immunology, AIDS Research Group, IDIBAPS, Hospital Clínic, University of Barcelona, Barcelona, Spain; 2 Centro Nacional de Biotecnología, CSIC, Madrid, Spain; 3 Infectious Diseases Unit, Hospital Clínic, IDIBAPS, University of Barcelona, Spain; 4 AIDS Immunopathogenesis Unit, Centro Nacional de Microbiología, Instituto de Salud Carlos III, Madrid, Spain; 5 Sección Inmunología, Laboratorio InmunoBiología Molecular, Hospital General Universitario Gregorio Marañón, Instituto de Investigación Sanitaria Gregorio Marañón (IISGM), Spanish HIV HGM Biobank, Networking Research Center on Bioengineering, Biomaterials & Nanomedicine (CIBERBBN), Madrid, Spain; Rush University, UNITED STATES

## Abstract

**Background:**

We have previously shown that an HIV vaccine regimen including three doses of HIV-modified vaccinia virus Ankara vector expressing HIV-1 antigens from clade B (MVA-B) was safe and elicited moderate and durable (1 year) T-cell and antibody responses in 75% and 95% of HIV-negative volunteers (*n* = 24), respectively (RISVAC02 study). Here, we describe the long-term durability of vaccine-induced responses and the safety and immunogenicity of an additional MVA-B boost.

**Methods:**

13 volunteers from the RISVAC02 trial were recruited to receive a fourth dose of MVA-B 4 years after the last immunization. End-points were safety, cellular and humoral immune responses to HIV-1 and vector antigens assessed by ELISPOT, intracellular cytokine staining (ICS) and ELISA performed before and 2, 4 and 12 weeks after receiving the boost.

**Results:**

Volunteers reported 64 adverse events (AEs), although none was a vaccine-related serious AE. After 4 years from the 1^st^ dose of the vaccine, only 2 volunteers maintained low HIV-specific T-cell responses. After the late MVA-B boost, a modest increase in IFN-γ T-cell responses, mainly directed against Env, was detected by ELISPOT in 5/13 (38%) volunteers. ICS confirmed similar results with 45% of volunteers showing that CD4+ T-cell responses were mainly directed against Env, whereas CD8+ T cell-responses were similarly distributed against Env, Gag and GPN. In terms of antibody responses, 23.1% of the vaccinees had detectable Env-specific binding antibodies 4 years after the last MVA-B immunization with a mean titer of 96.5. The late MVA-B boost significantly improved both the response rate (92.3%) and the magnitude of the systemic binding antibodies to gp120 (mean titer of 11460). HIV-1 neutralizing antibodies were also enhanced and detected in 77% of volunteers. Moreover, MVA vector-specific T cell and antibody responses were boosted in 80% and 100% of volunteers respectively.

**Conclusions:**

One boost of MVA-B four years after receiving 3 doses of the same vaccine was safe, induced moderate increases in HIV-specific T cell responses in 38% of volunteers but significantly boosted the binding and neutralizing antibody responses to HIV-1 and to the MVA vector.

**Trial registration:**

ClinicalTrials.gov NCT01923610.

## Introduction

Given the persistence of HIV epidemic, there is an urgent need to develop a safe and highly effective vaccine to control the HIV pandemic. To date only the RV144 phase III clinical trial using a combination of a recombinant canarypox vector vaccine (ALVAC-HIV [vCP1521]) plus a recombinant HIV-1 glycoprotein 120 (gp120) subunit vaccine (AIDSVAX B/E) had shown a moderate efficacy of 31.2% [1]. These results have highlighted that poxviruses should be considered as a suitable platform in the development of an HIV vaccine. Among the best studied vaccine vectors in humans are the poxviruses, particularly those strains with limited *in vivo* replicative capacity and, therefore, non-pathogenic in animal models and humans, such as MVA and NYVAC [2–5]. MVA is a highly attenuated vaccinia virus whose genome has lost about 30 kb of DNA including genes that counteract host immune responses [6]. MVA vectors expressing different HIV-1 antigens have been administered in homologous or heterologous combinations in humans to determine the safety, efficacy and immunogenicity profiles [3, 7, 8]. In general, MVA-based HIV vaccines have demonstrated to be safe but the immunogenicity observed has been quite heterogeneous. These differences depend on many parameters, such as the type and number of HIV-1 antigens expressed, the doses of vaccine used, the route of administration, the immunization protocol and the techniques used to analyze the vaccine-induced humoral and T cell responses [9].

We have previously shown, in a phase-I doubled-blind placebo-controlled trial (RISVAC02), that three doses of an MVA-vector expressing Env, Gag, Pol and Nef antigens from HIV-1 clade B (MVA-B) was safe, well tolerated and elicited moderate and durable HIV-specific T cell and antibody responses in 75% and 95% of healthy volunteers, respectively [10, 11]. In some infectious diseases, re-vaccination (single or multiple doses after several years) is recommended in order to boost the immune response and maintain the vaccine-induced protection [12] and it has been considered in the RV144 phase III clinical trial where a waning of efficacy was observed after 12 months of first dose of the vaccine [13]. Recent studies have applied this approach in MVA-based HIV vaccines that have been previously administered in healthy volunteers demonstrating enhanced humoral and cellular immunogenicity after the late boost [14, 15].

To extend these findings, we recruited 13 volunteers from the RISVAC02 trial to receive a fourth MVA-B boost (1×10^8^ pfu/dose) 4 years after the last immunization. We described here the safety and immunogenicity of administering a late MVA-B immunization and analyzed how this single dose impacts on the HIV- and vector specific T and B cell immune responses.

## Material and methods

### Subjects and samples

**Fig 1:**
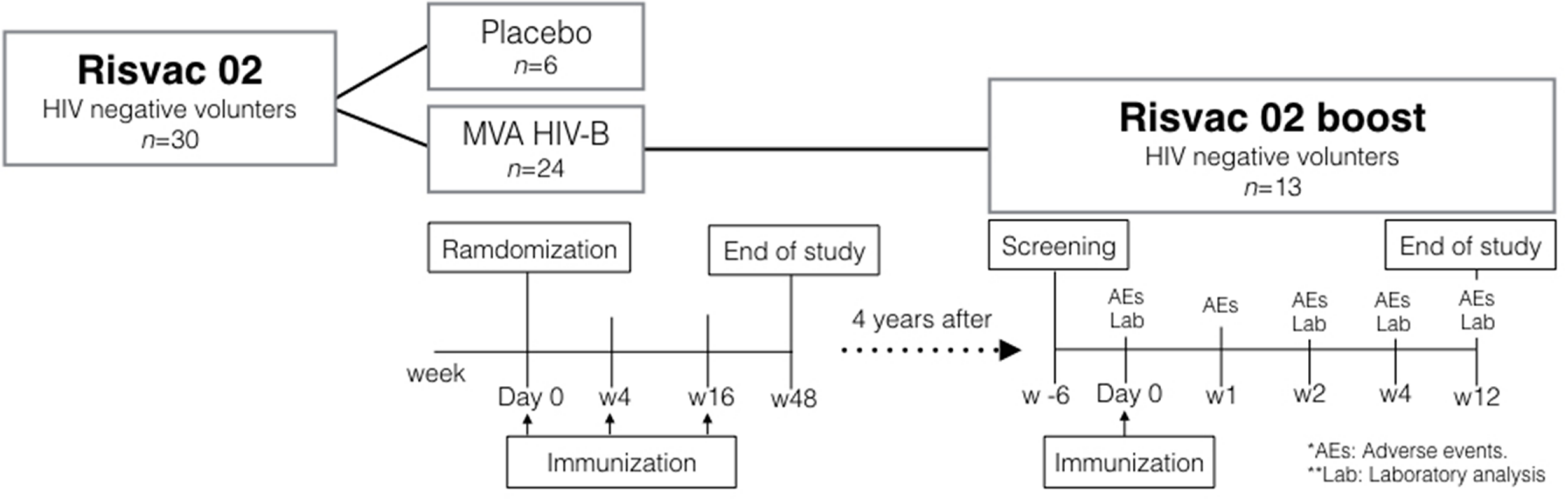
Disposition of participants and study flow chart. A total of 24 HIV-negative volunteers were vaccinated with MVA-B in the RISVAC02 study. Four years later, 13 of these volunteers were recruited to receive a single intramuscular boost of MVA-B vaccination in the RISVAC02boost clinical trial. Adverse Events (AEs), laboratory analyses and immunogenicity endpoints were tested as scheduled in the flow chart.

A phase I trial RISVAC02 was conducted during 2009 in 30 HIV-uninfected volunteers at low risk of HIV-1 infection that were randomly allocated to receive 3 intramuscular injections (1 × 10^8^ pfu/dose) of MVA-B (n = 24) or placebo (n = 6) at weeks 0, 4 and 16 [10]. All volunteers allocated to the vaccine arm (n = 24) were contacted again 4 years after the last immunization and, once it was verified that they continued to meet the same selection criteria (age between 18 and 55 years, at low risk of HIV-1 infection, no history of previous smallpox vaccination, and acceptance to use an effective method of contraception with partner from 14 days prior to the first vaccination until 4 months after the last immunization) established for the RISVAC02 trial, were offered to participate in a new study (RISVAC02boost). Thirteen out of 24 individual agreed to receive a late MVA-B boost, whereas 11 previous participants either declined, were not located or failed to meet, for various reasons, the inclusion criteria that they had previously met (e.g., pregnancy, HIV positive status, among others) “Fig 1”. The patients lost from RISVAC02 to RISVAC02boost studies are expected to be lost at random, and the loss pattern is not expected to be related to the previous treatment, neither its larger or smaller adverse effects. Note that the rejection criteria (not being located, getting pregnant, age, etc.) are not related to the previous treatment. However, the validity of our analysis is conditioned to the validity of this assumption. The study was explained to all volunteers in detail and all gave a written informed consent to receive one intramuscular injection (1 × 10^8^ pfu/dose) of MVA-B at week 0. Patient recruitment began on September 2013 and complete follow-up (until week 12 after the immunization) ended on December 2014. MVA-B was generated as described [16], and the Good Manufacturing Practices (GMP) lot was produced by IDT (Germany). The study was approved on April 15^th^ 2013 by the institutional ethical committees of the Hospital Clinic, Barcelona, Spain (Comitè Ètic d'Investigació Clínica), the Hospital Universitario Gregorio Marañón, Madrid, Spain (Comité Ético de Investigación Clínica) and by the Spanish Regulatory Authorities (AEMPS). The authors confirm that all ongoing and related trials for this drug/intervention were registered.

To evaluate the safety of administering a late dose of MVA-B we follow the same guideline used for the RISVAC02 trial [10]. The primary safety end-points were grade 3–4 local, systemic or laboratory adverse events (AEs), and secondary safety end-points were grade 1 and 2 AEs within 28 days of vaccination.

The primary immunogenicity end-points, quantitative or present/absent, were cellular T-cell responses (IFN-γ ELISPOT) at weeks 2, 4 and 12 following the immunization. Secondary immunogenicity end-points were T cell responses to HIV-1 and vaccinia virus (VACV)-specific antigens (intracellular cytokine staining (ICS) for CD4+ and CD8+ T cells), antibody responses (ELISA for antibodies reactive against HIV-1 Env and to the VACV vector and neutralizing antibody titters against HIV-1 and VACV before (week 0) and after weeks 2, 4 and 12 following the late MVA-B boost.

### Immunogenicity evaluations

#### ELISPOTS analysis

The immunogenicity of MVA-B was assessed on cryo-preserved peripheral blood mononuclear cells (PBMC) at weeks 0, 2, 4 and 12 after the immunization by the quantification of T-cell responses evaluated by a validated IFN-γ ELISPOT assay according to standardized operating procedures (SOP) in a single research laboratory following manufacturer’s instructions (BD Biosciences). Briefly, cryo-preserved PBMC were thawed and rested for 8 h at 37°C, and then 200,000 cells were stimulated with peptides pools (1μg of each single peptide) in 100μl of complete media (RPMI plus 10% FCS) in quadruplicate conditions. We used 8 pools of 50 to 61 overlapping peptides each (15 mers with 11 overlap) encompassing the Gag-Pol-Nef (GPN), and Env regions from clade B based on the sequence included in the virus vector. Media only was used as negative control. PHA-P (1μg/ml) and stimulation with CEF peptide pool were used as positive controls. Results are expressed as the mean number of spot forming cells (SFC)/10^6^ cells from quadruplicate assays. The following criteria were used to define the technical validity and positive responses: PBMC viability should be >80% to be analyzed; the assay background (media only) had to be <50 SFC/10^6^ PBMC; positive responses against PHA-P had to be above 500 SFC/10^6^ PBMC; and positive ELISPOT responses were considered when they were > 50 SFC/10^6^ PBMC and at least ≥ 3-fold over media control.

#### Flow cytometric assays (ICS assay)

Cryopreserved PBMC were thawed and rested overnight in RPMI 1640 medium containing 10% FCS. Then, PBMCs were stimulated for 6 h in complete RPMI 1640 medium containing 1μl/ml GolgiPlug (containing Brefeldin A; BD Biosciences), 0.7μl/ml GolgiStop (containing Monensin; BD Biosciences), 5 μg/ml anti-CD28 (BD Biosciences), anti-CD107a-FITC (BD Biosciences)and 1μg/ml each of the different HIV-1 peptide pools. For the detection of anti-vector response, PBMCs were stimulated as described above but using as stimulus autologous PBMCs infected with MVA at 2 pfu/cell for 16 h in a ratio of 2:1. At the end of the stimulation period, cells were washed, stained for the surface markers, fixed and permeabilized (Cytofix/Cytoperm Kit; BD Biosciences) and stained intracellularly using the appropriate fluorochromes. Dead cells were excluded using the Fixable Viability Stain 520 assay (BD Biosciences). The following fluorochrome-conjugated antibodies were used: CD3-PE-Cy7, CD4-Alexa 700, CD8-APC-H7, IFN-γ-PerCP-Cy5.5, IL-2-BV421 and TNF-α-APC for functional assays and CCR7-PE and CD45RA-PE-CF594 for phenotypic analysis. All antibodies were from BD Biosciences. Cells were acquired using a GALLIOS flow cytometer (Beckman Coulter). Analyses of the data were performed using FlowJo software version 10.0.7. (Tree Star, Ashland, OR). The average number of total events acquired was about 7 x 10^5^ cells. After gating, boolean combinations of single functional gates were then created using FlowJo software to determine the frequency of each response based on all possible combinations of cytokine expression or all possible combinations of differentiation markers expression. Background responses detected in negative control tubes (non-stimulated PBMCs) were subtracted from those detected in stimulated samples for every specific functional combination. An ICS was considered positive if the percentages of cytokine-positive cells in the stimulated samples were 3 times more than the values obtained in the unstimulated controls and if the background-subtracted magnitudes were higher than 0.02%.

### Antibody responses

Binding antibodies to Env and VACV proteins in serum, as well as neutralizing antibodies to VACV, were assessed at weeks 0, 2, 4, and 12 by ELISA and virus-plaque reduction assay according to SOP previously described [10, 16].

Sera were tested for neutralizing activity against a reporter virus carrying a Renilla luciferase gene in the place of *nef* and the BX08 full-length envelope [10]. Titrated recombinant viruses were preincubated with serial 4-fold dilutions of sera (1/20 to 1/327680) for 30 min at 37°C before the infection of the U87.CD4.CCR5 cells. Virus infectivity was determined 48 h post-inoculation by measuring luciferase activity in cell lysates using a 96-well plate luminometer (BioTek). Sigmoid curves were generated and ID50 and ID80 values were calculated by non-linear regression using GraphPad Prism software.

#### Statistical analysis

The safety endpoints were described and summarized by number, number per volunteer and percentage of adverse events (AEs), grading and location. We also divided the AEs into related (possibly, probably and definitely related to vaccination) and unrelated to vaccination (unlikely to be related, unrelated). The adverse events related to vaccination per volunteer at each three previous MVA-B immunizations (RISVAC02) were compared to boost results in the same 13 volunteers using the Wilcoxon signed-rank test.

The T-cell responses were analyzed as present or absent and reported as the number and proportion of participants responding to each peptide pool and for each time point. Median and Interquartile range (IQR) of magnitude of ELISPOT responses were described for each peptide pool and for each time point. The total ELISPOT responses were described as the sum of SFC of all positive responses, per peptide pool or after grouping pools from the same HIV protein, after subtraction of background.

To correct measurements of the medium response (RPMI) in the ICS analysis, we used a novel statistical approach previously described [17, 18]. Analysis and presentation of ICS results were performed by using SPICE version 5.1 software [19]. Comparisons of distributions were performed using a Student’s t test and a partial permutation test as previously described [19]. All values used for analyzing proportionate representation of responses are background-subtracted. For comparing the equality of proportion of responders between two groups we used the function proportionate test from [20].

The models used for the analyses were: log10(y) ~ x_patient + x_week + epsilon (for the analysis of humoral responses) (S2 Table) and y ~ x_patient + x_cellType + epsilon (for the analysis of frequency, function and phenotype of either HIV and VACV-specific T cell responses) (S3 Table); epsilon is supposed to be a random error, and the patient, week and cell type are supposed to be fixed effects.

## Results

### Clinical characteristics and safety

Thirteen healthy volunteers out of 24 (54.2%) who had received 3 doses of MVA-B during the RISVAC02 clinical trial agreed to receive a late boost of MVA-B and were invited to attend a screening visit. All volunteers who passed the screening received a single boost vaccine dose, given by intramuscular route, 4 years (range 4.1–4.7 years) after the last MVA-B immunization and completed the study as scheduled in “Fig 1”. The median age was 33 years (range 24–53 years) and the majority of them were males [12/13, (92.3%)]. Overall the vaccine was well tolerated. Volunteers reported 64 AEs, none of grade 3 or 4. Forty-nine (76.5%) AEs were related to vaccination (AEsRV), all of participants had at least one AEsRV, mostly were grade 1 (95.9%) and only 2 (4.1%) were grade 2. The median of total AEsRV per volunteer (AEsRV/v) (IQR) in RISVAC02boost was 3 (1–5.5), significantly higher than any other previous RISVAC02 immunization [w0: 2 (1–3.5), *p* = 0.044; w4: 1(0.5–3.5), *p* = 0.031; w16: 1(0.5–2), *p* = 0.003] [10]. This conclusion is valid as long as these differences are not due to regression to the mean effect and/or chronological bias.

Volunteer characteristics and AEs according to relationship of vaccination, distribution and gradation are shown in “Table 1” and AEsRV description and proportion of volunteers with side effects are summarized in “Table 2”.

**Table 1:** Volunteer characteristics and adverse events reported per volunteers according to relationship between vaccination, distribution and gradation.

c	Phase 1 HIV preventive trial MVA-B RISVAC02											RISVAC02boost MVA-B boost 4 years later
**Subjects’ characteristics**												
Volunteers *n*	24											13
Median age, years (IQR)	28 (22–35)											33 (27.5–38.5)
Male, *n*(%)	19 (79.2)											12 (92.3)
Volunteers completed all doses, *n* (%)	24 (100)											13(100)
**Adverse Events**	All vaccinated volunteers (*n* = 24)				Only RISVAC02 boost vaccinated volunteers (*n* = 13)*****							All vaccinated (*n* = 13)
*Time of immunization*	W0	W4	W16	Total	W0	W4	W16	Total	*p0*	*p4*	*p16*	W0
Total AEs *n*(%)	53(33)	61(39)	44(28)	158	31(39)	28(36)	20(25)	79	-	-	-	64
AEs* Characteristics												
AEs not related to vaccination** *n*(%)	10(32)	11(36)	10(32)	31(20)	4(37)	4(37)	3(26)	11(14)	-	-	-	15(24)
AEs related to vaccination*** *n*(%)	43(34)	50(39)	34(27)	127(80)	27(40)	24(35)	17(25)	68(86)	-	-	-	49(76)
AEsRV/v**** median (IQR)	2(1–2)	1(1–3.7)	1(1–2)	4(2.2–9)	2(1–3.5)	1(0.5–3.5)	1(0.5–2)	4(2–9.5)	0.044	0.031	0.003	3(1–5.5)
*Distribution (only AEsRV)*												
Local *n*(% vs Systemic)		24(56)	25(50)	21(62)	70(55)	13(48)	11(46)	11(65)	35(51)	-	-	-	19(39)
Local/v median (IQR)		1(1–1)	1(0.2–1.7)	1(0–1)	3(2–4)	1(0.5–1.5)	1(0–1.5)	1(0–1)	2(1.5–4)	0.058	0.013	0.004	1(1–2)
Systemic *n(% vs Local)*		19(44)	25(50)	13(38)	57(45)	14(52)	13(54)	6(35)	33(49)	-	-	-	30(61)
Systemic/v median (IQR)		0(0–1)	0(0–2)	0(0–1)	1(0–4.5)	0(0–2)	0(0–2)	0(0–1)	1(0–5.5)	0.111	0.083	0.026	1(0–4)
*Intensity (only AEsRV)*												
Grade 1 *n*(% vs grade 2)		40(93)	48(96)	34(100)	122(96)	25(93)	22(92)	17(100)	64(94)	-	-	-	47(96)
Grade 1/v median (IQR)		1.5(1–2)	1(1–3.7)	1(1–2)	3.5(2–7.5)	2(0.5–3.5)	1(0.5–3.5)	1(0.5–2)	4(2–8.5)	0.032	0.020	0.003	3(1–5.5)
Grade 2 *n*(% vs grade 1)		3(7)	2(4)	0(0)	5(4)	2(7)	2(8)	0(0)	4(6)	-	-	-	2(4)
Grade 2/v median (IQR)		0(0–0)	0(0–0)	0(0–0)	0(0–0)	0(0–0)	0(0–0)	0(0–0)	0(0–0.5)	1.000	1.000	0.317	0(0–0)
Grade ≥3 *n*(%)		0(0)	0(0)	0(0)	0(0)	0(0)	0(0)	0(0)	0(0)	-	-	-	0(0)
Days to AEsRV median (IQR)	1(0–5)	1(0–2)	0(0–1.2)	1(0–2)	1(1–5)	0.5(0–1)	0(0–3.5)	1(0–2)	0.118	0.237	0.865	0(0–2)
Days to AEsRV resolution median (IQR)	2(1–3)	2(1–3)	2.5(1–4)	2(1–3)	1(1–3)	2(1–3)	2(1–3)	2(1–3)	0.016	0.021	0.028	1(0–2)

Volunteer characteristics and adverse events reported per volunteers according to relationship between vaccination, distribution and gradation. Also shows previous RISVAC02 results for all volunteers and the thirteen that subsequently participated in the RISVAC02boost. Adverse events related to vaccination after MVA-B boost with each MVA-B previous immunization compared in the same patients using the Wilcoxon signed-rank test.

*AEs: Adverse events.

**AEs not related to vaccination: not related and unlikely to be related to vaccination are included.

***AEsRV: AEs related to vaccination. Definitely, probably or possibly related to vaccination are included.

****/v: per volunteer. IQR: interquartile range.

*****p0, p4, p16: p-value when comparing Risvac02 boost results and W0, W4 and W16 Risvac02 previous patient data respectively.

**Table 2:** Adverse events related to vaccination description and proportion of volunteers suffering any side effects.

Adverse events related to vaccination description				
Trial	RISVAC02 Only RISVAC02boost vaccinated volunteers			RISVAC02boost MVA-B boost 4 years later
	w0	w4	w6	
Volunteers	*n* = 13	*n* = 13	*n* = 13	*n* = 13
**Adverse events related to vaccination (*AEsRV*)** *n AEsRV* [*n* (%) volunteers]				
Any type of AEsRV*	27 [11(85)]	24 [10(77)]	17 [10(77)]	49 [13(100)]
Local AEsRV	13 [10(77)]	11 [8(62)]	11 [9(69)]	19 [13(100)]
Pain	12 [10(77)]	6 [6(46)]	8 [8(62)]	14 [13(100)]
Itching	0 [0(0)]	2 [1(8)]	3 [2(15)]	3 [2(15)]
Redness	1 [1(8)]	3 [2(15)]	0 [0(0)]	2 [2(15)]
Induration	0 [0(0)]	0 [0(0)]	0 [0(0)]	0 [0(0)]
Intensity local AEsRV				
Grade 1	11 [9(69)]	10 [8(62)]	11 [9(69)]	19 [13(100)]
Grade 2	2 [2(15)]	1 [1(8)]	0 [0(0)]	0 [0(0)]
Systemic AEsRV	14 [6(46)]	13 [6(46)]	6 [5(38)]	30 [8(61)]
Malaise	6 [5(38)]	5 [5(38)]	3 [3(23)]	7 [7(54)]
Myalgia	2 [1(8)]	2 [2(15)]	1 [1(8)]	6 [6(46)]
Chills	0 [0(0)]	0 [0(0)]	0 [0(0)]	6 [6(46)]
Headache	5 [4(31)]	4 [4(31)	1 [1(8)]	5 [4(31)]
Fever	0 [0(0)]	0 [0(0)]	0 [0(0)]	4 [4(31)]
Flu-like syndrome	1 [1(8)]	1 [1(8)]	0 [0(0)]	1 [1(8)]
Nausea/vomiting	0 [0(0)]	1 [1(8)]	0 [0(0)]	1 [1(8)]
Intensity systemic AEsRV				
Grade 1	14 [6(46)]	12 [6(46)]	6 [5(38)]	28 [8(61)]
Grade 2	0 [0(0)]	1 [1(8)]	0 [0(0)]	2 [1(8)]

*AEsRV: AEs related to vaccination. Definitely, probably or possibly related to vaccination are included.

### Cellular immunogenicity

#### HIV-1-specific T cell responses

Ten out of 13 volunteers who were revaccinated had shown cellular responses in the first clinical trial (RISVAC02). On the day of the late MVA-B boost, 2 out of these 10 volunteers (20%) maintained positive HIV-specific T cell responses, although at a lower level. At week 2 after the boost, HIV-1 specific immune responses were detected in 5 volunteers (38%) by ELISPOT. This frequency of responders was maintained until the end of the follow-up at week 12. Conversely, 5 volunteers who showed positive T cell responses at RISVAC02 study did not show any positive HIV-specific T cell response after the MVA-B boost. Moreover, 3 vaccinees did not respond neither at the first regimen of immunizations nor after MVA-B late boost.

**Fig 2:**
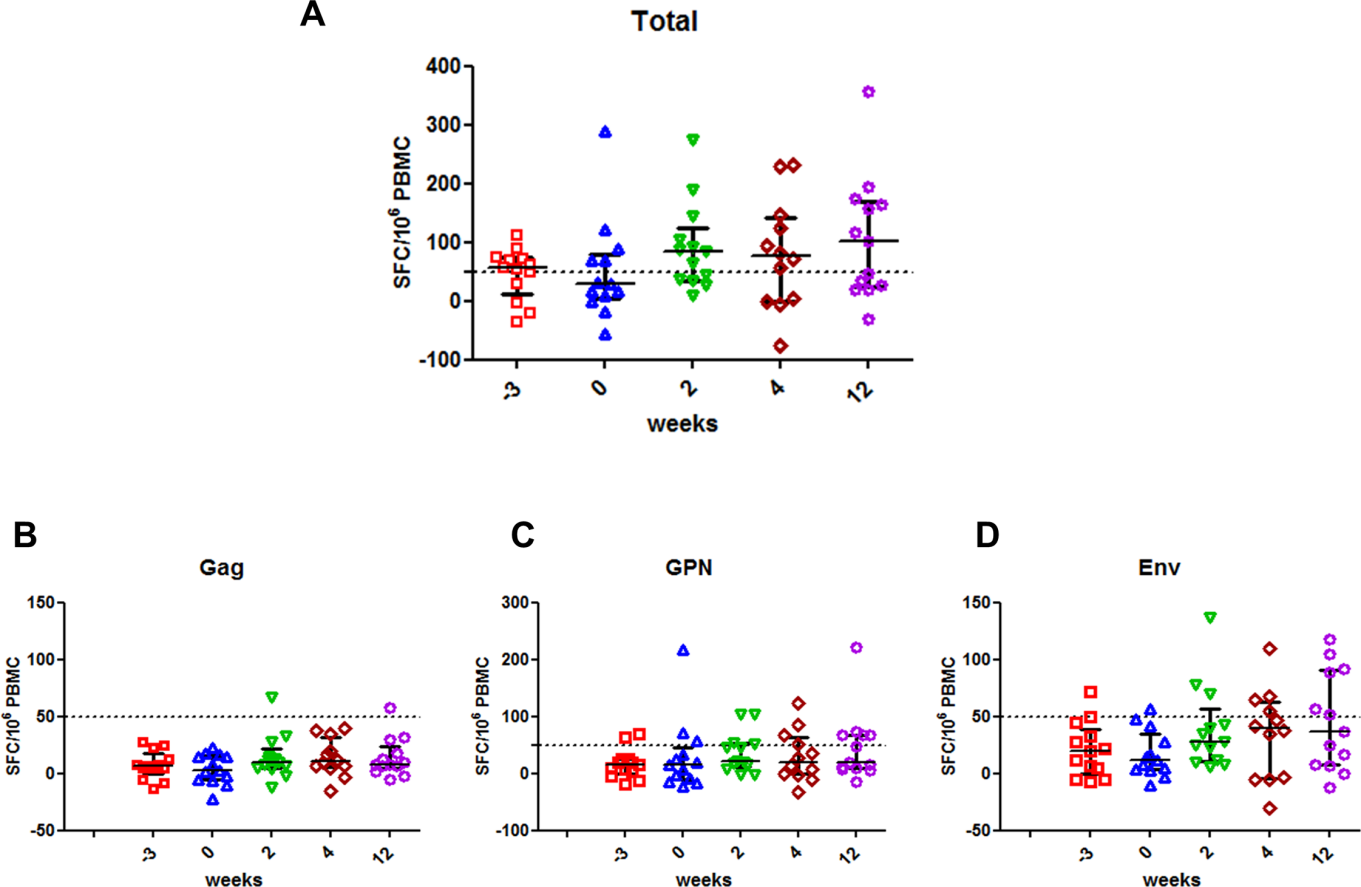
ELISPOT results. Magnitude of HIV-1-specific T cell responses measured by IFN-γ-based ELISPOT is shown. A) Total responses, represented as the sum of positive responses to Gag, GPN and Env peptide pools; B) T-cell responses to Gag peptide pools; C) Positive responses to GPN peptide pools, and D) T-cell responses to Env peptide pools. The graphs show the frequency of HIV-1-specific T cell responses by SFC/106 PBMC at different time-points (-3, w0, w2, w4 and w12). Week -3 corresponds to w48 of follow-up of RISVAC02 clinical trial. Median and IQR are represented in all the graphs for the different time-points evaluated.

Concerning the magnitude of the HIV-specific T cell responses, the median and IQR at w0 was 28 (4–78) SFC/10^6^ PBMC. A modest increase in the magnitude of the T cell responses was observed in 5/13 (38%) volunteers at the different time-points assayed (median and IQR: 83 (35–125) (p = 0.090), 77 (0–141) (p = 0.420) and 102 (23.5–168) (p = 0.090) SFC/10^6^ PBMC at w2, w4 and w12, respectively) “Fig 2A”. Vaccine induced T-cell responses were predominantly directed against Env and Gag-Pol-Nef (GPN) peptide pools, as it was observed in RISVAC02 trial “Fig 2B, 2C and 2D”.

Next we assessed the frequency and functional profile of the HIV-specific CD4 and CD8 T cell responses elicited before and after the late MVA-B boost in 11 volunteers by polychromatic ICS as previously described [11].

**Fig 3:**
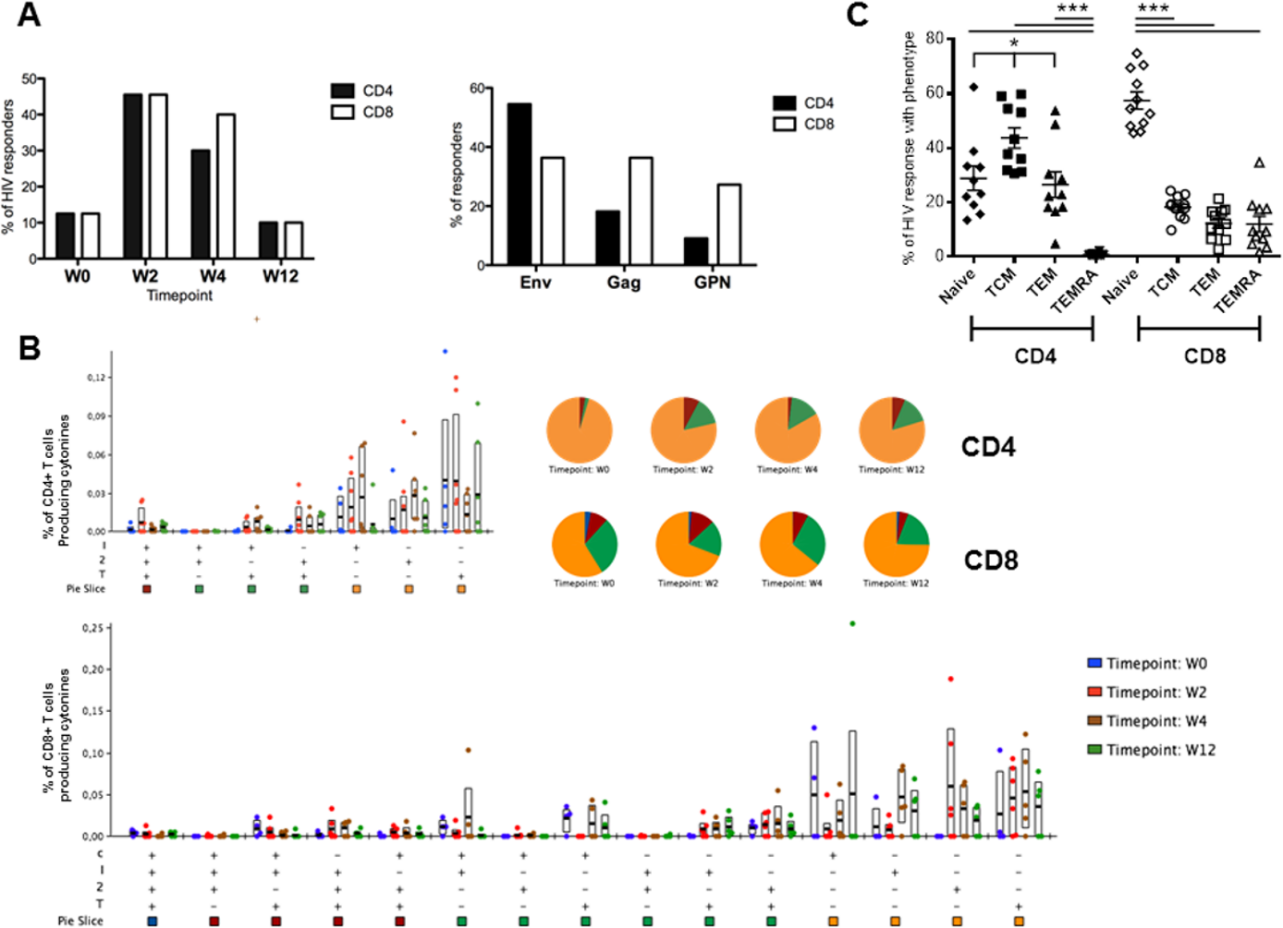
Frequency, function and phenotype of HIV-specific T cell responses. A: Percentage of responders with positive ICS responses against Env+Gag+GPN at the different time points (left panel) and distribution of the CD4+ and CD8+ T cells by antigen (right panel). B: Functional profile of vaccine-induced T cells. The quality of the HIV-specific CD4 or CD8 T cell response is characterized by the proportion of cells making every possible combination of the measured cytokines: IFN-γ (I); IL-2 (2); TNF-α (T) and CD107a (C). Responses are grouped and colour coded on the basis of the number of functions. The bar charts show the mean values and interquartile ranges (IQR) and the pie charts show the average proportion of the HIV-specific CD4 or CD8 T cell responses according to the functions at weeks 0, 2, 4 and 12. “+”distributions that are different from the earliest time point (W0) within each category at p<0.05 (Student's T test). C: Phenotype of vaccine-induced T cells. The graphic represents the distribution of the responding HIV-specific CD4 and CD8 T cells at any time point based on CCR7 expression in combination with CD45RA within the Naïve (CD45RA+ CCR7+), T central memory (TCM: CD45RA- CCR7+), T effector memory (TEM: CD45RA- CCR7-) or terminally differentiated T effector memory (TEMRA: CD45RA+ CCR7-) phenotypes. Statistical differences were determined using ANOVA test (using the linear model y ~ x_patient + x_cellType + epsilon) followed by Tukey's honest significant difference criterion. *p<0.05, ***p<0.005.

After four years of the last MVA-B immunization (w0) only 12.5% of volunteers maintained CD4+ and CD8+ T cell responses against HIV-1 antigens (Env+Gag+GPN). However, at w2 and w4 after the late MVA-B boost we detected HIV-specific CD4 and CD8 T cells in about 45% of the volunteers. In all the time points assayed the magnitude of the responses were low (range 0.06–0.15) and there were no significant differences between them (p>0.05). The frequency of responders decreased to baseline values at w12. The CD4+ T cell response was essentially directed against the Env peptide pool at all time-points, whereas the CD8+ T cell response was more evenly distributed between Env, Gag and GPN antigens “Fig 3A”. The functional profile of the HIV-specific CD4 and CD8 T cell responses was analyzed in the responding volunteers and we found that activated T cells expressed predominantly a monofunctional profile in both lineages, without significant changes during the follow-up, although about 20% and 35% of CD4+ and CD8+ T cells respectively exhibit two or three functions “Fig 3B”. There were no significant changes during the follow-up in the polyfunctionality of the responses (p>0.05).

We also characterized the differentiation stages of the responding HIV-specific CD4 and CD8 T cells at any time-point “Fig 3C” into T central memory (TCM: CD45RA- CCR7+), T effector memory (TEM: CD45RA- CCR7-), naïve T cells (CD45RA+ CCR7+) or terminally differentiated T effector memory (TEMRA: CD45RA+ CCR7-) populations as previously described [21]. The phenotype of CD4+ T cell responses was preferentially of TCM whereas naïve was the most abundant phenotype for CD8+ T cells. A supplementary S2 Table shows the differences between groups, confidence intervals and p-values for each of the comparisons.

#### VACV-specific T cell responses

**Fig 4:**
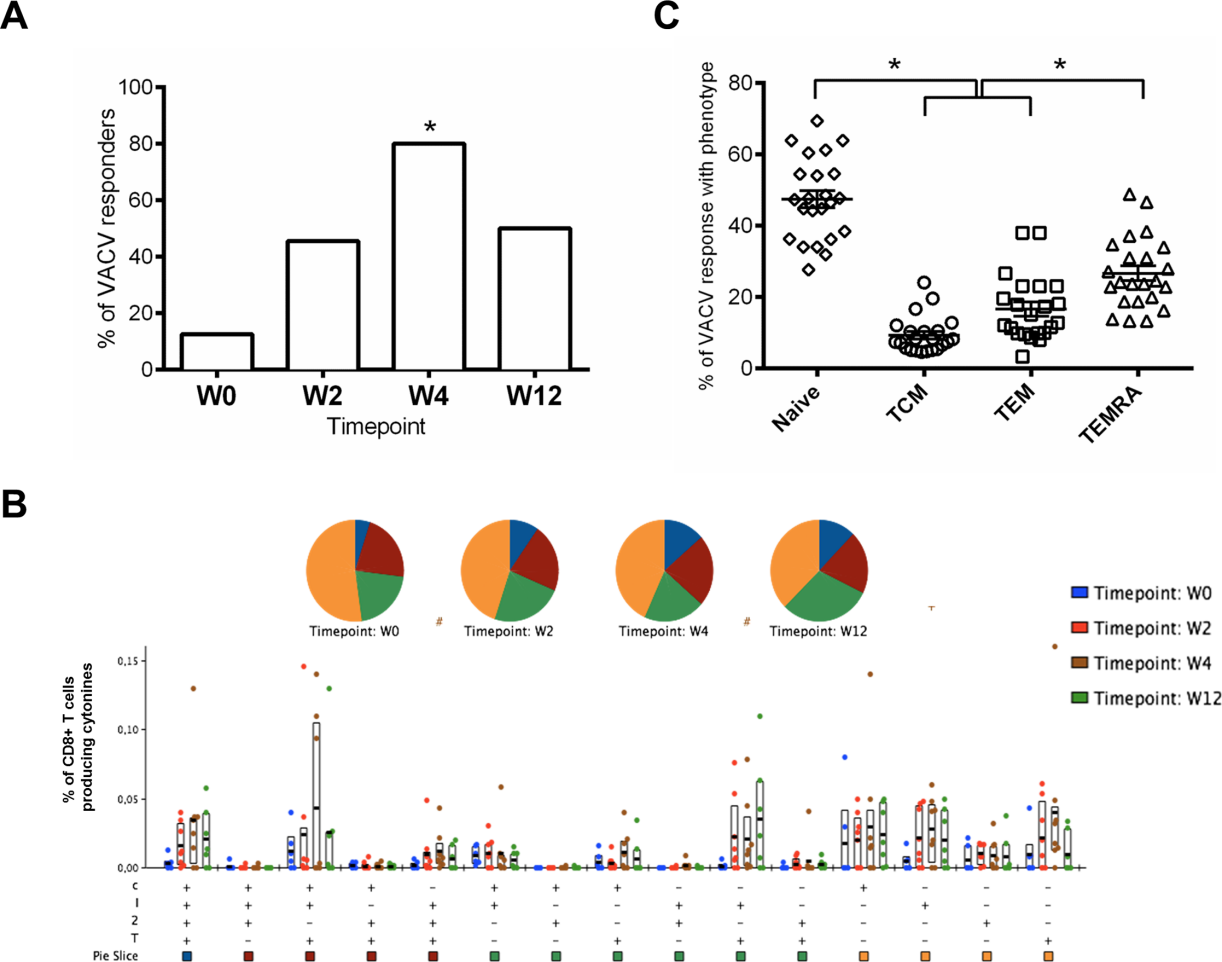
Frequency, function and phenotype of VACV-specific CD8 T cell responses. A: Percentage of responders with positive ICS responses against MVA infected cells at the different time points. The equality of proportion between groups was determined using the function prop.test. *p = 0.017. B: Functional profile of VACV-specific CD8 T cells. The quality of the VACV-specific CD8 T cell response is characterized by the proportion of cells making every possible combination of the measured cytokines: IFN-γ (I); IL-2 (2); TNF-α (T) and CD107a (C). Responses are grouped and colour coded on the basis of the number of functions. The bar charts show the mean values and interquartile ranges (IQR) and the pie charts show the average proportion of the VACV-specific CD8 T cell responses according to the functions at weeks 0, 2, 4 and 12. “+”distributions that are different from the earliest time point (W0) within each category at p<0.05 using Student's T test or “#” Wilcoxon signed rank test. C: Phenotype of VACV-specific CD8 T cells. The graphic represents the distribution of the VACV-specific CD8 T cells at any time point based on CCR7 expression in combination with CD45RA within the Naïve (CD45RA+ CCR7+), T central memory (TCM: CD45RA- CCR7+), T effector memory (TEM: CD45RA- CCR7-) or terminally differentiated T effector memory (TEMRA: CD45RA+ CCR7-) phenotypes. Statistical differences were determined using ANOVA test (using the linear model y ~ x_patient + x_cellType + epsilon) followed by Tukey's honest significant difference criterion. *p<0.05.

At 4 years after the last MVA-B immunization (w0) 12.5% of vaccinees maintained CD8+ T cell responses against VACV antigens; however, the response rates increased with time after the late MVA-B boost and peaked at w4, with 80% of responders (p = 0.017). The magnitudes of the responses were similar in all the time points assayed (range 0.05–0.406) with no significant differences (p>0.05). At w12 the frequency of responders decreased to 50% “Fig 4A”.

The VACV-specific T cell responses were highly polyfunctional, with about 60% of MVA-specific CD8 T cells displaying more than one function “Fig 4B” and with a phenotype distributed mainly within the naïve and TEMRA populations “Fig 4C”.

### Humoral immunogenicity

#### Binding antibodies to HIV-1 gp120

**Fig 5:**
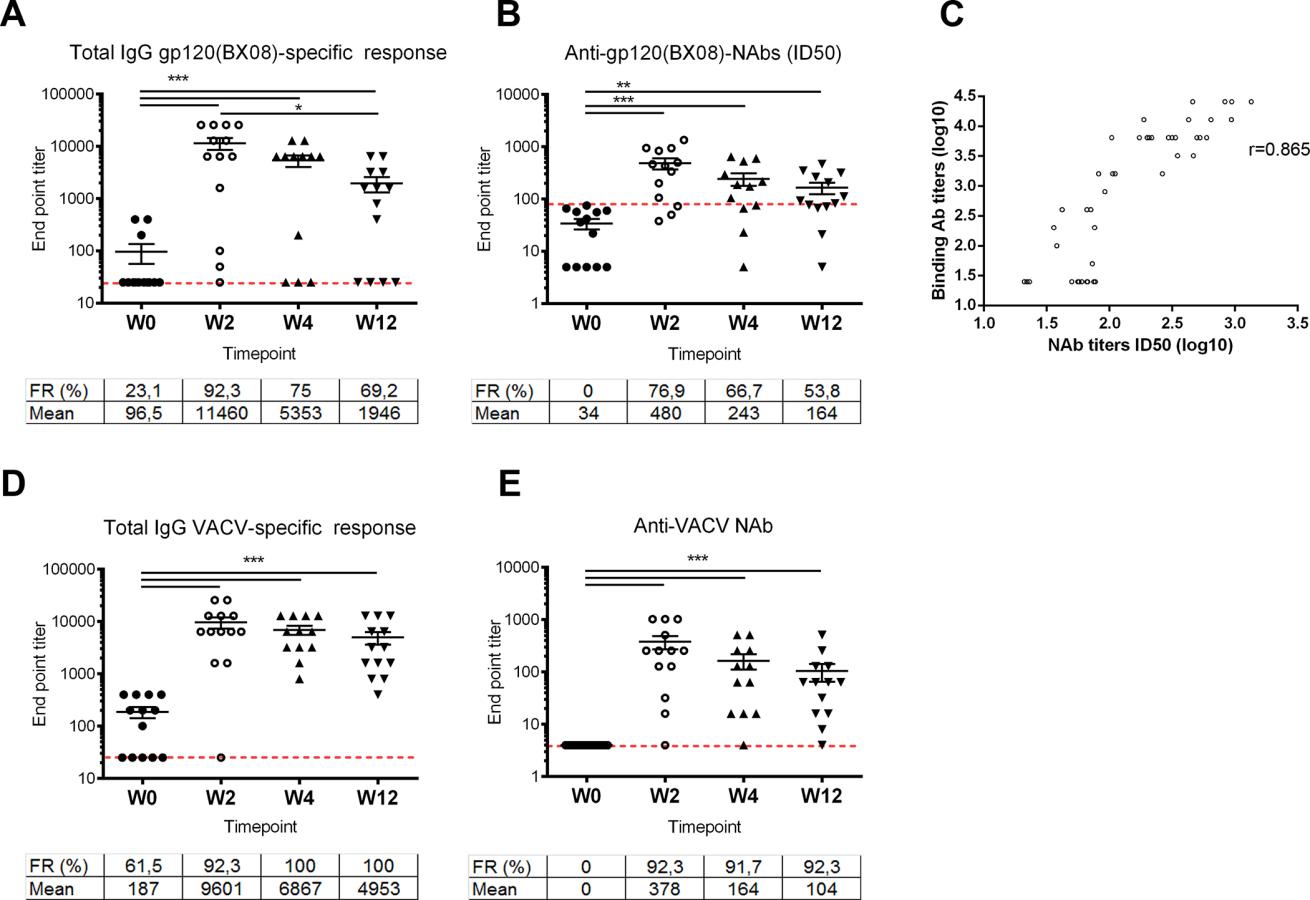
Humoral responses. A: Total IgG binding antibody titers against HIV-1 gp120 (BX08). B: BX08 neutralization ID50 titers. C: Correlation between BX08 binding IgG and neutralizing ID50 titers. D: Total IgG binding titers against VACV proteins. E: VACV neutralization ID50 titers. The frequency of responders and the mean titers at the different time points are shown in each graph. Dashed line represents the threshold considered as positive response. Statistical differences were evaluated by one way ANOVA test (using the linear model log10(y) ~ x_patient + x_week + epsilon) followed by Tukey's honest significant difference criterion. *p<0.05, **p<0.01, *** p<0.005 (***4). Pearson’s correlation coefficient (r value) was calculated between BX08 binding IgG and neutralizing ID50 titers.

Fig 5A shows that before the immunization (w0) 3 out of 13 volunteers (23.1%) were reactive by ELISA with a mean titer of 96.5 (considering all the vaccinees). However, MVA-B boost significantly enhanced the response rates and the titer of binding antibodies to HIV-1 gp120 (from isolate Bx08). The HIV-antibody responses peaked 2 weeks after the boost with a mean titer of 11460, and decline overtime to mean titers of 5353 and 1946 at w4 and w12, respectively. The frequency rate also peaked at w2 with 92.3% of responders falling to 75% and 69.2% at w4 and w12, respectively. (S1 Table)

#### Anti-vaccinia virus antibodies

We also evaluated the levels of total IgG antibodies against vaccinia virus proteins over time in the serum of vaccine recipients. As shown in Fig 5B, a high proportion of volunteers 61,5% (8/13) still maintain anti-VACV antibodies with a mean titer of 187 before the late MVA-B boost (4 years after the last immunization). However, as for HIV-1 Env, after MVA-B boost there was a significant enhancement of both, the response rates and the titer of VACV-specific antibodies. The VACV-antibody responses peaked 2 weeks after the boost with a mean titer of 9601, and decline overtime to mean titers of 6867 and 4953 at w4 and w12 respectively. The frequency rate peaked at w4 with 100% of responders and was maintained until the end of the follow-up (w12) (S1 Table).

The anti-VACV neutralizing antibody (Nab) responses were also determined (S1 Table). As shown in Fig 5C, before the MVA-B boost none of the volunteers had detectable levels of anti-VACV Nabs. The anti-vector NAb responses peaked 2 weeks after the boost with a mean NAb titer of 378, and decline overtime to mean NAb titers of 164 and 104 at w4 and w12 respectively. Moreover, the frequency of responders (over 90%) behaved similarly after the boost in all of the time points assayed. A supplementary S3 Table shows the differences between groups, confidence intervals and p-values for each of the comparisons.

#### HIV-1 neutralizing antibodies

Serum neutralizing activity against HIV-1 BX08 virus was measured at weeks 2, 4 and 12 after receiving the late boost. As it is shown in “Table 3”, no positive responses were observed at w0 while 10 out of 13 volunteers (77%) were able to neutralize HIV-1 after 2 weeks of immunization with a titer > 1/90 (that we consider as positive). Thereafter, we observed a decrease of the neutralizing activity titers until the end of the follow-up (w12).

**Table 3:** Serum neutralizing activity against HIV-1 BX08 virus at 2, 4 and 12 weeks after receiving the boost. Data are shown as the reciprocal dilution giving 50% and 80% neutralization (ID50 and ID80 titers). Reciprocal serum ID50 and ID80 values ≥1000 are highlighted in red, ≥400 and <1000 in orange, ≥200 and <400 in dark yellow, ≥90 and <200 in light yellow, and <90 in white. The given reciprocal titers correspond to 1/dilution of serum.

	Week 0		Week 2		Week 4		Week 12	
Serum	ID50	ID80	ID50	ID80	ID50	ID80	ID50	ID80
**203**	<90	<90	428	103	217	<90	111	<90
**204**	<90	<90	461	107	188	<90	<90	<90
**205**	<90	<90	336	<90	527	<90	207	<90
**206**	<90	<90	106	<90	<90	<90	<90	<90
**210**	<90	<90	832	197	173	<90	<90	<90
**211**	<90	<90	<90	<90	<90	<90	<90	<90
**215**	<90	<90	1348	462	645	230	316	137
**115**	<90	<90	200	<90	104	<90	<90	<90
**101**	<90	<90	943	349	n.d.	n.d.	467	146
**102**	<90	<90	936	356	595	155	349	107
**103**	<90	<90	498	196	295	113	264	95
**104**	<90	<90	<90	<90	<90	<90	<90	<90
**105**	<90	<90	<90	<90	<90	<90	<90	<90

## Discussion

We have previously reported that three doses of a recombinant modified vaccinia virus Ankara vector expressing HIV-1 antigens Env, Gag, Pol, and Nef from clade B (MVA-B) administered to 24 HIV-negative volunteers, was safe, well tolerated and immunogenic [10, 11]. Here we extended our previous results and explored the safety and immunogenicity of a fourth MVA-B boost delivered 4 years after the last immunization.

Regarding safety, all volunteers that received the vaccine suffered at least one side effect related to vaccination, although most were considered as grade 1 (96%) and no grade 3 or 4 were reported. However, the number of side effect per volunteer was significantly higher than in any of the 3 first immunizations given 4 years before, probably related to an anamnestic reaction to the vaccine. Despite these data, MVA vector had a good safety profile when administered in late boost.

In terms of immunogenicity, we show that, similarly to other studies that evaluated the persistence of T cell immune responses using MVA-based vectors [14, 22, 23], after 4 years of being vaccinated with MVA-B, only a small proportion of individuals maintained low HIV-specific T cell responses when measured by ELISPOT (20%) or ICS (12,5% for each T cell subset). This suggests that 3 doses of MVA-B did not induced long-term T cell memory against HIV infection [22]. In fact, when we analyzed the impact of a fourth MVA-B boost, we detected a very modest increase in the response rates by ELISPOT and ICS in only a 38% and 45% of volunteers, respectively. Interestingly, the activated CD8+ T cells after the late MVA-B boost had a naïve phenotype, based on the differential expression of CD45RA and CCR7, indicating that the homologous immunization regimen might not induce high frequencies of long-term memory T cells against HIV infection. Other studies that evaluated the impact of a late boost of an MVA vector expressing HIV antigens [14] or adjuvanted HIV Env protein [15] on the T cell immune responses reported higher response rates and magnitudes. However, the boost was administered in volunteers that previously received a heterologous DNA prime/ MVA boost approach.

The durability of the humoral immune responses was an important issue addressed in our study. In some clinical trials, a decrease of 3 to 10 fold in the magnitude of Env-specific antibodies six months after vaccination has been reported [1, 24]. Here we observed at w0 that the magnitude of the Env-specific binding antibodies was 10 times lower compared to the obtained in the RISVAC02 trial at the peak of the response (2 weeks after the third MVA-B dose (w18)), but it was similar to the detected at eight months after the last immunization. However, systemic binding antibodies to gp120 Bx08 significantly increased in most volunteers after a late boost with MVA-B and reached titers of a median 11460. This mean titer was 10-fold higher compared to the detected at w18 in the RISVAC02 trial or other studies [7, 10, 14], and was in the same range as that reported in either the RV144 trial [1, 13] and other studies [23, 24]. These data highlight the ability of the MVA-B vector to establish long-lived antibody responses to HIV-1 Env, probably providing survival signals in responding B cells that allow them to expand efficiently after a late boost. Moreover, a late boost of MVA-B was able to induce HIV-1 neutralizing activity of serum in more than 75% of individuals. The rate of responders and the antibodies titer were higher when compared to the observed during RISVAC02 trial [10]. This also reinforces that responses could be related to the induction of B-cell memory by previous MVA-B immunizations. When we performed a correlation coefficient, we found a strong correlation between binding antibodies to gp120 and the HIV neutralizing titers “Fig 5”, suggesting that further increases in antibody levels of MVA-B, like boosting with optimized purified HIV-1 Env (gp140) trimmers, might improve the neutralization capacity of the homologous immunization protocol.

Finally, we observed in volunteers vaccinated with MVA-B 4 years ago, that 12.5% of the vaccinees had VACV-specific CD8 T cell responses, indicating that the anti-vector responses elicited by 3 doses of MVA-B were of limited duration. After booster, the CD8+ T cell response rate increased to 80% (w4), while the magnitudes were similar at the different time points assayed. For antibody responses to the vector, only 20% maintained anti-VACV antibodies, while after the boost with MVA-B, 100% of the volunteers developed anti-vector antibodies at w4, and over 90% had neutralizing activity against VACV. These data support the findings of previous studies suggesting that pre-existing immunity to MVA did not reduce the proportion of individuals who responded to HIV-1, but did lower the magnitude of responses [24].

In summary, our results show that one boost of MVA-B four years after receiving 3 doses of the same vaccine against HIV was safe, with more reactogenicity than previous immunizations. The late MVA-B boost induced moderate increases in the HIV-specific T cell responses but significantly boosted the antibody responses to HIV-1 Env protein as well as the generation of HIV-1 neutralizing antibodies. Hence, MVA-based vaccines have the potential to be further explored as a suitable component of an optimal HIV vaccine regimen.

## Supporting information

S1 TREND Statement Checklist (PDF)

S1 Table Humoral immune response induced in patients by the late MVA-B boost. Values of serum antibody reactivity by ELISA against g120 (BX08) and vaccinia virus (VACV), as well as neutralizing antibody titers to VACV are shown.
(DOC)

S2 Table Results of the statistical test performed with data represented in “Fig 3C”. Differences between groups, confidence intervals and p-values for each of the comparisons are shown.
(DOCX)

S3 Table Results of the statistical test performed with data represented in “Fig 5A, 5B and 5C”. Differences between groups, confidence intervals and p-values for each of the comparisons are shown.
(DOCX)

S1 Protocol RISVac02 boost (EudraCT: 2013-000635-27). (DOC)
