# Correction: Safety and vaccine-induced HIV-1 immune responses in healthy volunteers following a late MVA-B boost 4 years after the last immunization

**DOI:** 10.1371/journal.pone.0195915

**Published:** 2018-04-10

**Authors:** Alberto C. Guardo, Carmen Elena Gómez, Vicens Díaz-Brito, Judit Pich, Joan Albert Arnaiz, Beatriz Perdiguero, Juan García-Arriaza, Nuria González, Carlos O. S. Sorzano, Laura Jiménez, José Luis Jiménez, María Ángeles Muñoz-Fernández, José M Gatell, José Alcamí, Mariano Esteban, Juan Carlos López Bernaldo de Quirós, Felipe García, Montserrat Plana, for the RISVAC02boost study

The first author is listed incorrectly in the citation. Please view the correct citation here:

Guardo AC, Gómez CE, Díaz-Brito V, Pich J, Arnaiz JA, Perdiguero B, et al. (2017) Safety and vaccine-induced HIV-1 immune responses in healthy volunteers following a late MVA-B boost 4 years after the last immunization. PLoS ONE 12(10): e0186602. https://doi.org/10.1371/journal.pone.0186602
