# Supplementary material for: Safety and vaccine-induced HIV-1 immune responses in healthy volunteers following a late MVA-B boost 4 years after the last immunization
Source: PLoS One. 2017 Oct 24;12(10):e0186602. doi: 10.1371/journal.pone.0186602 (PMC5655491; doi:10.1371/journal.pone.0186602)
Supplement: S1 Table — Values of serum antibody reactivity by ELISA against g120 (BX08) and vaccinia virus (VACV), as well as neutralizing antibody titers to VACV are shown. (DOC) [file pone.0186602.s002.doc]

|  | **Total IgG gp120-specific response** | | | |  | **Total IgG VACV-specific response** | | | |  | **Anti VACV-NAb** | | | |
| --- | --- | --- | --- | --- | --- | --- | --- | --- | --- | --- | --- | --- | --- | --- |
|  | **Week 0** | **Week 2** | **Week 4** | **Week 12** |  | **Week 0** | **Week 2** | **Week 4** | **Week 12** |  | **Week 0** | **Week 2** | **Week 4** | **Week 12** |
|  | <50 | 12800 | 6400 | 1600 |  | 400 | 6400 | 3200 | 6400 |  | <1/8 | 256 | 128 | 64 |
|  | <50 | 25600 | 12800 | 800 |  | <50 | 6400 | 6400 | 1600 |  | <1/8 | 256 | 64 | 64 |
|  | <50 | 6400 | 6400 | 6400 |  | 400 | 6400 | 12800 | 12800 |  | <1/8 | 256 | 256 | 128 |
|  | <50 | 1600 | 200 | <50 |  | 200 | 6400 | 6400 | 1600 |  | <1/8 | 128 | 128 | 32 |
|  | <50 | 25600 | 6400 | 400 |  | <50 | 25600 | 6400 | 1600 |  | <1/8 | 256 | 64 | 64 |
|  | <50 | 100 | <50 | <50 |  | <50 | 6400 | 3200 | 800 |  | <1/8 | 128 | 16 | 16 |
|  | 400 | 25600 | 12800 | 6400 |  | 200 | 25600 | 12800 | 12800 |  | <1/8 | 1024 | 512 | 256 |
|  | <50 | 6400 | 6400 | 1600 |  | <50 | 1600 | 1600 | 800 |  | <1/8 | 32 | 16 | 16 |
|  | <50 | 25600 | ND | 3200 |  | 100 | 12800 | ND | 3200 |  | <1/8 | 512 | ND | 128 |
|  | 200 | 12800 | 6400 | 3200 |  | 400 | 12800 | 12800 | 12800 |  | <1/8 | 1024 | 512 | 512 |
|  | 400 | 6400 | 6400 | 1600 |  | 200 | 12800 | 12800 | 6400 |  | <1/8 | 1024 | 256 | 64 |
|  | <50 | 50 | <50 | <50 |  | 400 | 1600 | 3200 | 3200 |  | <1/8 | 16 | 16 | 8 |
|  | <50 | <50 | <50 | <50 |  | <50 | <50 | 800 | 400 |  | <1/8 | <1/8 | <1/8 | <1/8 |
| **Mean** | **96,2** | **11460** | **5356** | **1946** |  | **187** | **9602** | **6867** | **4954** |  | **0** | **378** | **164** | **104** |
| **Std. Deviation** | 143 | 10675 | 4565 | 2267 |  | 163 | 8234 | 4732 | 4865 |  | 0 | 392 | 184 | 141 |
| **Std. Error of Mean** | 39,7 | 2961 | 1318 | 629 |  | 45,3 | 2284 | 1366 | 1349 |  | 0 | 109 | 53,2 | 39 |

.

**S1 Table:** Humoral immune response induced in patients by the late MVA-B boost. Values of serum antibody reactivity by ELISA against g120 (BX08) and vaccinia virus (VACV), as well as neutralizing antibody titers to VACV are shown.
