# Supplementary material for: Safety and vaccine-induced HIV-1 immune responses in healthy volunteers following a late MVA-B boost 4 years after the last immunization
Source: PLoS One. 2017 Oct 24;12(10):e0186602. doi: 10.1371/journal.pone.0186602 (PMC5655491; doi:10.1371/journal.pone.0186602)
Supplement: S2 Table — Differences between groups, confidence intervals and p-values for each of the comparisons are shown. (DOCX) [file pone.0186602.s003.docx]

**Supplementary table S2**: Results of the statistical test performed with data represented in Fig 3C

CD4

diff lwr upr p adj

TEM-Naive 14.80 -2.02464 31.6246404 0.0997018

TCM-Naive -12.39 -29.21464 -0.4346404 0.0497963 *

TEMRA-Naive -28.12 -44.94464 -11.2953596 0.0005258 ***

TCM-TEM -17.19 -34.01464 -0.3653596 0.0438748 *

TEMRA-TEM -42.92 -59.74464 -26.0953596 0.0000010 ***

TEMRA-TCM -25.73 -42.55464 -8.9053596 0.0014588 **

CD8

diff lwr upr p adj

TEM-Naive -39.608 -51.27932 -27.936678 0.0000000 ***

TCM-Naive -45.493 -57.16432 -33.821678 0.0000000 ***

TEMRA-Naive -46.637 -58.30832 -34.965678 0.0000000 ***

TCM-TEM -5.885 -17.55632 5.786322 0.5223020

TEMRA-TEM -7.029 -18.70032 4.642322 0.3698872

TEMRA-TCM -1.144 -12.81532 10.527322 0.9930872
