# Supplementary material for: Safety and vaccine-induced HIV-1 immune responses in healthy volunteers following a late MVA-B boost 4 years after the last immunization
Source: PLoS One. 2017 Oct 24;12(10):e0186602. doi: 10.1371/journal.pone.0186602 (PMC5655491; doi:10.1371/journal.pone.0186602)
Supplement: S3 Table — Differences between groups, confidence intervals and p-values for each of the comparisons are shown. (DOCX) [file pone.0186602.s004.docx]

**Supplementary table S3:** Results of the statistical test performed with data represented in Fig 5A, B and C

Anti-gp120 (Fig 5A)

| diff lwr upr p adj | | | | |
| --- | --- | --- | --- | --- |
| W2-W0 2,9310005 1,8062726 4,05572831 0,0000003 *** | | | | |
| W4-W0 2,0989635 0,9742357 3,22369139 0,0000910 *** | | | | |
| W12-W0 1,8210897 0,6963618 2,94581755 0,0006270 *** | | | | |
| W4-W2 -0,8320369 -1,8808510 0,21677721 0,1599432 | | | | |
| W12-W2 -1,1099108 -2,1587249 -0,06109663 0,0347609 * | | | | |
| W12-W4 -0,2778738 -1,3266880 0,77094029 0,8897673 | | | | |
|  |  |  |  |  |
| Anti-VACV (Fig 5B) |  |  |  |  |
| diff lwr upr p adj |  |  |  |  |
| W2-W0 2,09737844 1,1707177 3,0240392 0,0000030 *** | | | | |
| W4-W0 1,93528537 1,0086246 2,8619461 0,0000128 *** | | | | |
| W12-W0 1,97335075 1,0466900 2,9000115 0,0000091 *** | | | | |
| W4-W2 -0,16209307 -1,0887538 0,7645677 0,9649355 | | | | |
| W12-W2 -0,12402769 -1,0506884 0,8026331 0,9837054 | | | | |
| W12-W4 0,03806539 -0,8885954 0,9647261 0,9995061 | | | | |
|  |  |  |  |  |
| Anti-VACV NAb (Fig 5C) |  |  |  |  |
|  |  |  |  |  |
| diff lwr upr p adj | | | | |
| W2-W0 2,17667843 1,638287 2,715070299 0,0000000 *** | | | | |
| W4-W0 1,66724305 1,128851 2,205634922 0,0000000 *** | | | | |
| W12-W0 1,64408690 1,105695 2,182478768 0,0000000 *** | | | | |
| W4-W2 -0,50943538 -1,047827 0,028956491 0,0692243 | | | | |
| W12-W2 -0,53259153 -1,070983 0,005800338 0,0534245 | | | | |
| W12-W4 -0,02315615 -0,561548 0,515235715 0,9994333 | | | | |
