# Supplementary material for: Safety and vaccine-induced HIV-1 immune responses in healthy volunteers following a late MVA-B boost 4 years after the last immunization
Source: PLoS One. 2017 Oct 24;12(10):e0186602. doi: 10.1371/journal.pone.0186602 (PMC5655491; doi:10.1371/journal.pone.0186602)
Supplement: S1 Protocol — (DOC) [file pone.0186602.s005.doc]

**RisVac 02boost**

**Safety and immunological response of a boosting dose of MVA-B in healthy volunteers after 4 years of receiving MVA-B**

**The trial will be conducted at Hospital Clinic**

Principal Investigator Dr. Felipe García fgarcia@clinic.ub.es

00 34 932275586

Clinical Investigator Dr Agathe León aleon@clinic.ub.es

Infectious Diseases Unit

Hospital Clinic

Villarroel, 170

08036 Barcelona, Spain Fax: 00 34 934514438

**and at the Hospital Gregorio Marañón**

Principal Investigator Dr. Juan Carlos López Bernaldo de Quirós

jclopez@mx3.redestb.es

00 34 915868565

Clinical Investigator Dr. Cecilia Martínez Fernández-Llamazares

cmartinezf.hgugm@salud.madrid.org

00 34915290405

Servicio de Farmacia

Hospital Gregorio Marañón

C/DR ESQUERDO Nº46

28007 MADRID, Spain

Fax: 00 34 915866621

**The trial will be coordinated by the Hospital Clinic Clinical Trials Unit**

Investigators Dr. Joan Albert Arnaiz jaarnaiz@clinic.ub.es

00 34 932275400 (ext 2815)

Dr Judit Pich jpich@clinic.ub.es

00 34 932275400 (ext 2815)

Hospital Clinic Clinical Trials Unit

Villarroel, 170

08036 Barcelona, Spain Fax: 00 34 932279877

**MVA B will be supplied by**

Impfstoffwerk Dessau-Tornau GmbH (IDT)

Streetzer Weg 15a

Rodleben OT Torna

D-06862

Germany

**The trial will be sponsored by**

Fundació Clínic per a la Recerca Biomèdica

Dr. Josep M Gatell. Infectious Diseases Unit gatell0@attglobal.net

Hospital Clínic 00 34932275430

Villarroel, 170 Fax: 00 34 934514438

08036 Barcelona, Spain

**Investigations will be conducted in the following Immunology Laboratories**

**Cellular responses (Elispot and flow cytometry):**

Dr. Montserrat Plana mplana@ub.edu

00 34 932275400 (ext 2884)

AIDS Research Laboratory

Hospital Clinic

Villarroel, 170

08036 Barcelona, Spain Fax: 00 34 934514438

**Antibody responses**

Dr Mariano Esteban

[mesteban@cnb.uam.es](mailto:mesteban@cnb.uam.es)

00 34 915854553

Departamento de Biología Molecular y Celular

Centro Nacional de Biotecnología

Campus Universidad Autónoma. Cantoblanco.

28049-Madrid. Spain

Fax: 00 34 915866621

**Version 1.0, 14^th^ January 2013**

**EudraCT: 2013-000635-27**

[I. Introduction 5](#__RefHeading___Toc348103265)

[II Summary of Trial 10](#__RefHeading___Toc348103266)

[III Allocation of immunisations 12](#__RefHeading___Toc348103267)

[IV Scientific questions to be addressed and analyses: 12](#__RefHeading___Toc348103268)

[1. General plan 13](#__RefHeading___Toc348103269)

[1.1 Objectives 13](#__RefHeading___Toc348103270)

[1.2 Design 13](#__RefHeading___Toc348103271)

[1.3 Population. 13](#__RefHeading___Toc348103272)

[1.4 Trial products 14](#__RefHeading___Toc348103273)

[1.5 End-points 15](#__RefHeading___Toc348103274)

[2 Schedule of Visits 16](#__RefHeading___Toc348103275)

[2.1 Recruitment 16](#__RefHeading___Toc348103276)

[2.2 Screening 16](#__RefHeading___Toc348103277)

[2.3 Trial Entry 17](#__RefHeading___Toc348103278)

[2.4 Follow-up visits 17](#__RefHeading___Toc348103279)

[2.5 Final visit 17](#__RefHeading___Toc348103280)

[2.6 Study schedule 19](#__RefHeading___Toc348103281)

[3. Procedures 20](#__RefHeading___Toc348103282)

[3.1 Blood and urine collection 20](#__RefHeading___Toc348103283)

[3.2 Clinical history and examination 20](#__RefHeading___Toc348103284)

[3.3 Genital infection screen 20](#__RefHeading___Toc348103285)

[3.4 Pre-HIV test screen and counselling 20](#__RefHeading___Toc348103286)

[3.5 Safe sex counselling and condom provision 20](#__RefHeading___Toc348103287)

[3.6 HIV related issues 20](#__RefHeading___Toc348103288)

[3.7 Discontinuation procedures (including withdrawal) 21](#__RefHeading___Toc348103289)

[4. Assessments 21](#__RefHeading___Toc348103290)

[4.1 Safety assessments 21](#__RefHeading___Toc348103291)

[4.2 Immunogenicity assessments 22](#__RefHeading___Toc348103292)

[4.3 Other assessments 23](#__RefHeading___Toc348103293)

[5. Adverse events 23](#__RefHeading___Toc348103294)

[5.1 Definitions 23](#__RefHeading___Toc348103295)

[5.2 Relationship to study product 24](#__RefHeading___Toc348103296)

[5.3 Reporting adverse events 24](#__RefHeading___Toc348103297)

[5.4 Clinical management 25](#__RefHeading___Toc348103298)

[6 Management of HIV issues during and following the trial 25](#__RefHeading___Toc348103299)

[6.1 HIV testing 25](#__RefHeading___Toc348103300)

[6.2 HIV infection 25](#__RefHeading___Toc348103301)

[6.3 Social discrimination as a result of a post-vaccine response 26](#__RefHeading___Toc348103302)

[7. Management of the trial 26](#__RefHeading___Toc348103303)

[7.1 Data management at the Clinical Centres 26](#__RefHeading___Toc348103304)

[7.2 Data management in the immunology laboratories 27](#__RefHeading___Toc348103305)

[7.3 Data management at the Hospital Clinic CTU 27](#__RefHeading___Toc348103306)

[7.4 Monitoring by HOSPITAL CLÍNIC CTU 27](#__RefHeading___Toc348103307)

[7.5 Data Ownership 28](#__RefHeading___Toc348103308)

[7.6 Trial Coordinating Committee (TCC) 28](#__RefHeading___Toc348103309)

[7.7 Data and Safety Monitoring Committee (DSMC) 28](#__RefHeading___Toc348103310)

[8 Statistical considerations 28](#__RefHeading___Toc348103311)

[8.1 Sample size 28](#__RefHeading___Toc348103312)

[8.2 Analysis 29](#__RefHeading___Toc348103313)

[9 Confidentiality, ethics and responsibilities, including indemnity 29](#__RefHeading___Toc348103314)

[10 Publication 30](#__RefHeading___Toc348103315)

[11 References: 31](#__RefHeading___Toc348103316)

# I. Introduction

Given the relentless persistence of the HIV epidemic in the face of anti-retroviral therapy, there is an urgent need to develop preventative approaches which will limit HIV transmission. Recently, advances in HIV prevention via preexposure prophylaxis [1], topical microbicides [2] and a weakly effective prime-boost vaccine [3] have been reported. Despite these advances, a safe and potent vaccine remains the most powerful tool for epidemic control of infectious diseases. A phase IIb clinical trial (the STEP study) using serotype 5 adenovirus expressing HIV-1 Gag/Pol/Nef failed to demonstrate prevention of HIV-1 infection or reduction of early viral load [4, 5]. Conversely, a combination of a recombinant canarypox vector vaccine (ALVAC-HIV [vCP1521]) plus a recombinant HIV-1 glycoprotein 120 (gp120) subunit vaccine (AIDSVAX B/E) had an efficacy of 31.2% [3]. While this last trial showed only a modest benefit, the results offer insight for future research and have renewed interest in the development of poxviruses as vectors capable of stimulating the cellular and humoral arms of the immune response [6].

The best studied vaccine vectors in humans are the pox viruses. Particular attention has been, however, focused on pox viruses with limited *in vivo* replicative capacity and, therefore, non pathogenic in animal models and humans [7, 8]. The modified vaccinia virus Ankara (MVA) strain has deletions of the genes associated with pathogenicity. During extensive field studies, including high risk patients, no side effects were associated with the use of the MVA vaccine [7, 9]. MVA vectors expressing different HIV-1 genes have been tested in phase I clinical trials in humans. These have appeared to be safe and well tolerated [10-12]. However, limited immunogenicity has been usually reported when MVA is used alone [13-16] [11, 12]. This low level of response is similar to other poxvirus used alone [17] [18] [19]. On the other hand, recent phase I/II clinical trials using either MVA [20] or NYVAC (derived from the Copenhagen strain of vaccinia virus) [21] have shown that poxvirus-based HIV-1 vaccine candidates are highly immunogenic in prime–boost immunization regimens with DNA.

**MVA-B**

MVA

The Modified Vaccinia virus Ankara (MVA) has been developed towards the end of the smallpox eradication campaign in the seventies of the previous century, to obtain a vaccine with a better safety profile than the vaccines that were in use at that time. MVA was derived from Vaccinia strain Ankara, by over 570 passages in chicken embryo fibroblast cells (CEF) [22]. The complete genomic sequence has been sequenced and has a length of 178 kb. It consists of 193 open reading frames (ORFs), corresponding to 177 genes, of which 25 are split and/or have suffered mutations resulting in truncated proteins. These numerous mutations, affecting host interactive proteins and some structural proteins, explain the attenuated phenotype of MVA[8].

The resulting MVA strain had lost the capacity to productively infect mammalian cells and suffered six major deletions of DNA, totaling 31,000 base pairs. MVA was proven to be avirulent even in immunosuppressed animals. There is clinical experience with MVA as vaccine against smallpox in over 120,000 people[7, 9]. During extensive field studies, including high risk patients, no side effects were associated with the use of the MVA vaccine[7, 9]. The combination of a very good safety profile and the ability to deliver antigens in a highly immunogenic way makes MVA suitable as vaccine vector. MVA can stimulate antibody and T cell responses even in presence to pre-existing antibodies[23]. MVA has shown to be effective in primates and humans for several viruses, including SIV and SHIV[10, 24-27].

***Inserts of MVA-B***

The HIV genes expressed in the recombinant vector are derived from the HIV-1 natural isolate BX08 (for gp120) and Gag-Pol-Nef from HIV-IIIB. All HIV genes have been optimised for codon usage since it has recently been shown that humanisation of synthetic HIV gene codons allowed for an enhanced and REV/RRE- independent expression of *env* and *gag-pol* genes in mammalian cells. Genes were optimised for both safety and translation efficiency.

The *env* gene has been designed to express the secreted gp120 form of the envelope proteins and contain an optimal synthetic leader sequence for enhanced expression. The *gag, pol* and *nef* genes were fused to express a Gag-Pol-Nef polyprotein. An artificial -1 frameshift introduced in the natural slippery sequence of the *p7-p6* gene junction results in an in-frame Gag-Pol-Nef fusion protein due to the absence of ribosomal frameshift. An N-terminal Gly → Ala substitution in *gag* prevents the formation and release of virus-like particles from transfected cells. This strategy should allow for an equimolar production of Gag, Pol and Nef proteins and an enhanced MHC Class-I restricted presentation of their CTL epitopes.

For safety and regulatory reason, the packaging signal sequence has been removed; protease active site mutated; the integrase gene deleted; and the reverse transcriptase gene disrupted by insertion of a scrambled *nef* gene at the 3’ end of the DNA sequence coding for the RT active site known to be associated with an immunodominant CTL epitope. The *nef* gene has been dislocated by fusing its 5’ half to its 3’ half without losing its immunodominant CTL epitopes.

Plasmids pMA60gp120B/gagpolnefB-12,17 and pLZAW1 were provided by Sanofi-Pasteur. A 5.6 kbp DNA fragment containing the two synthetic early/late (E/L) promoters in a back-to-back orientation individually driving a codon optimized _BX08_gp120 and _IIIB_GPN genes of HIV-1 clade B was excised from plasmid pMA60gp120B/gagpolnefB-12,17 with the restriction endonuclease BamHI. The insert was modified by incubation with Klenow DNA polymerase to generate blunt ends, and inserted into the pLZAW1 vector (previously digested with AscI, modified by incubation with Klenow, and dephosphorylated by incubation with Alkaline Phosphatase, Calf Intestinal (CIP) generating the plasmid transfer vector pLZAW1gp120B/gagpolnef-B-1. The resulting plasmid directs the insertion of the foreign genes into the TK locus of MVA genome and allows the generation of a recombinant virus without selectable marker.

**Construction of the recombinant virus MVA-B**

Primary chick embryo fibroblast cells (CEF) from 11-day old SPF eggs were infected with MVA at a multiplicity of 0.05 PFU/cell and then transfected with 10 μg DNA of plasmid pLZAW1gp120B/gagpolnef-B-1 using lipofectamine reagent according to the manufacturer´s protocol (Invitrogen, San Diego, CA). After 72 h post infection the cells were harvested, sonicated and used for recombinant virus screening. Recombinant MVA viruses containing the _BX08_gp120 and _IIIB_Gag-Pol-Nef genes from clade B, and transiently co-expressing the β-gal marker gene (MVA-B (X-gal+)), were selected by consecutive rounds of plaque purification in CEF cells stained with 5-bromo-4-chloro-3-indolyl β-galactoside (X-Gal) (300 μg/mL). In the following plaque purification steps, recombinant MVA viruses containing the _BX08_gp120 and _IIIB_Gag-Pol-Nef genes and having deleted the β-gal gene (by homologous recombination between the TK left arm and the short TK left arm repeat that are flanking the marker) were isolated by two additional consecutive rounds of plaque purification screening for non-staining viral foci in CEF cells in the presence of X-Gal (300 μg/mL). In each round of purification the isolated plaques were expanded in CEF cells for 3 days, and the crude virus obtained were used for the next plaque purification round. The resulting MVA-B virus was grown in CEF, purified through two 45% (w/v) sucrose cushions and titrated by immunostaining. Purity of the recombinant virus was confirmed by PCR with primers spanning the junction and internal regions of the inserts and by DNA sequence analysis.

The multigenic recombinant MVA-B (env, gag, pol, nef clade C) has been shown to be stable for the HIV genes after 10 passages.

**Non-clinical studies with HIV-1 specific recombinant MVA-B vaccines**

***Toxicity and biodistribution***

The MVA-B vaccine used in this clinical study has been tested for toxicity and biodistribution in rats (unpublished study report by Convance Laboratories Ltd, Harrogate, UK). In this study 40 rats (20 male and 20 female) received three intramuscular administrations (three doses at 14 day intervals) of 0.4 mL vaccine (0.2 mL into each calf muscle), 1x10^8^ pfu/mL, which far exceeds the equivalent dose that will be used in this phase-1 clinical study. A control group was injected with the same volume of saline on the same time points. Of each group, ten animals were necropsied on Day 30 and ten animals were necropsied on day 51 (of each 10 5 were examined for toxicity and 5 for PCR on MVA-B).

There were no unscheduled deaths after administration of MVA-B. There were no treatment‑related clinical signs. Body weight and food consumption were unaffected by treatment. No effect of treatment was detected by the ophthalmoscopic examinations. Increases in neutrophils of 2.3 and 4.3 fold were recorded for treated males and females respectively on day 30 (24 hours after completion of the third dosing). By day 51 (22 days after the final dose) the vaccinated animals had circulating levels of neutrophils similar to controls. There was no effect of treatment on clinical chemistry parameters. Organ weights were unaffected by treatment.

At the day 30 kill, there were no macroscopic findings due to either local or systemic effects of the MVA B HIV vaccine. Microscopically, there was a minor increase in the severity of inflammatory cell foci/ myositis at the injection sites of treated animals compared with controls, suggestive of a minor local effect due to the MVA-B vaccine. There were no microscopic findings suggestive of systemic effects due to the MVA B HIV vaccine. At the day 51 kill, there was evidence of almost complete reversal of the injection site findings seen in treated animals at the day 30 kill. In other tissues in treated animals, there were no microscopic findings suggestive of delayed systemic effects due to the MVA B HIV vaccine.

Furthermore, other HIV-1 specific recombinant MVA vaccines have been evaluated for safety and biodistribution in animal studies. Biodistribution and safety of the HIV-1 specific recombinant MVA vaccine MVA-HIVA expressing HIV-1 clade A *gag* p24/p17 sequences (the same vaccine as used in the clinical trial cited below [28] was examined in rhesus macaques infected with SIV (SIVmac32H or SIVmac220 ) and in SCID (severe combined immunodeficiency) mice [29]. The macaques were vaccinated with a single dose of 5 x 10^7^ pfu intradermally, and the mice with 5 x 10^6^ pfu intradermally on days 0 and 15. In the macaques, after intradermal administration of MVA·HIVA no organ tropism was detected that could correlate with target organ toxicity, since no MVA-HIVA DNA could be detected in any of the examined organs (testes, epididymis, ovary, blood, brain, heart, spleen, kidney, liver, mesenteric lymph nodes, draining axillary lymph nodes, skin and muscle at injection site). In particular, no positive signal was found in any gonad sample from male or female animals, so the risk of transfer of vaccine DNA into the germ cells using this route of administration is regarded to be negligible.

Similarly to SIV-infected rhesus macaques, intradermal administration of MVA·HIVA into severely immunodeficient mice lacking T and B cell responses has not posed a significant safety concern nor it increased a risk for persistence of the vaccine-derived nucleic acid sequences. A positive PCR signal in the vaccinated SCID mice was observed only at the earliest examined timepoint, 49 days after the second vaccination, in 4 out of 6 skin samples at the injection site. This results are consistent with earlier results with the same vaccine in non-immunodeficient mice [30] and the safety results of MVA in severely immunocompromized cynomolgus macaques [31].

***Immunogenicity and challenge data in animal models***

Mice inoculated with MVA-B in prime/boost protocols with the homologous vector or with heterologous vector, like DNA-B (two plasmid vectors that express either gp120 or Gag-Pol-Nef, same inserts as for MVA-B) induced specific immune responses against the four HIV antigens in both Balb/c and humanized HLA-A2 mice [32]. Moreover, a similar MVA construct but expressing the HIV-1 89.6p (gp120) and the poliprotein Gag-Pol-Nef of SIVmac239 induced in macaques specific immune responses to the four HIV-SIV immunogenes and trigger high and long-term protection following challenge with pathogenic SHIV89.6p [33].

There is other efficacy data of several animal studies with other HIV-1 or SIV specific recombinant MVA vaccines. The key studies with SIV specific recombinant MVA vaccines in macaques are summarized in table 1. No safety problems were reported in these studies, although only the first study [34] explicitly mentioned the absence of side effects after vaccination with MVA. These studies reported CTL responses [24, 35, 36], neutralizing antibody responses [24, 37, 38], lower plasma SIV RNA levels[24, 34, 36, 37] and even prolonged survival[37] in vaccinated animals.

**Clinical studies with MVA-B vaccine**

We have recently reported the results of the first-in-man phase-I, doubled blind placebo-controlled trial in healthy volunteers that was performed to investigate the safety and immunogenicity of this HIV/AIDS vaccine candidate [39][40]. Previous studies in human dendritic cells infected with MVA-B revealed that the inserted HIV-1 genes induced the expression of cytokines, cytokine receptors, chemokines, chemokine receptors and molecules involved in antigen uptake and processing, including the major histocompatibility complex genes, that might act as regulators of immune responses to HIV-1 antigens [41].

A total of 30 HIV-uninfected male and female volunteers at low risk of HIV-1 infection, and with absence of smallpox specific antibodies and no history of previous smallpox vaccination were included in 2 Spanish Clinical Centers in Madrid and Barcelona. The study was explained to all subjects in detail, and all gave a written informed consent and were randomly allocated to receive 3 intramuscular injections (1x10^8^ pfu/dose) of MVA-B (n=24) or placebo (n=6) at weeks 0, 4 and 16. All volunteers were followed until week 48.

***Safety***

30 participants were included in the safety analysis.The median age was 27 years (range 19-48). Twenty-four (80%) were male and 27 Spanish (90%).

Overall the vaccine was well tolerated. A total of 169 adverse events (AE) were reported during follow-up (158 in vaccinated and 11 in placebo group). One hundred and forty-five AE were grade 1, 8 grade 2 and 5 grade 3. Fifty-two of grade 1-2 AE were considered as definitely related to vaccination (4 out of 8 grade 2 AE were considered as definitely related). There were 5 AE grade 3 in 3 volunteers (all in vaccinated group), but none was considered related to vaccination (1 volunteer had tonsillitis, 1 volunteer had a traffic accident and 1 volunteer had 1 pneumonia and 2 asthmatic attacks). The number of AE considered as definitely or probably related to vaccination decreased progressively from the first to third vaccination.

The most frequently reported local reactogenicity AE was pain that appeared in all vaccinees, although only in 1 subject was grade 2. The most frequently reported systemic reactogenicity events were headache and malaise, and both of them were grade 1. The median (IQR) duration of local AE was 3days (IQR 2-3), the systemic AE 1.5 days (IQR 1-2). There were no laboratory changes considered as grade 3 or 4.

*Immunogenicity*

*Cellular immunoresponses.* Overall 75% of the vaccinated volunteers responded, as measured by ELISPOT, to any pool included in the vector at any time point. Positive T-cell responses responses were detected by ELISPOT in 58%, 67%, 50% and 68% of vaccinees, at weeks 6, 8, 18 and 20, respectively. The proportion of responders increased clearly after the second dose of vaccine from 33% of responders at week 4 (second dose) to 67% at week 8. At week 16 (third dose) the proportion of responders decreased to 50% to increase again up to 68% at week 20. Vaccine-specific responses were maintained at least until week 48 in 15/22 (68%) individuals.

Regarding the magnitude of the response, the median of the total responses induced was 236, 139, 288 and 156 SFC/10^6^ PBMC at weeks 6, 8, 18 and 20, respectively and was similar against HIV-1 Gag, GPN and Env pools. The peak of the magnitude of the total response was at week 4 with a median of 474 SFC/10^6^ PBMC (range 193-589). At this time, after the first immunization, the greatest magnitude was against Env pools (median: 209, range: 123-399 SFC/10^6^ PBMC). By contrast, we observed that the higher responses against Gag and GPN were observed at week 18, after the third immunization (medians: 239 and 143 SFC/10^6^ PMBC, respectively). At week 48, the magnitude of the total response decreased to a median of 132 SFC/10^6^ PBMC (range 52-690) .

*Binding antibodies to gp160.* 11 out of 24 volunteers (45.8%), 23 out of 24 (95.8%), and 16 out of 22 (72.7%) formed binding antibodies against HIV-1 subtype B gp160 at weeks 8, 18 and 48, respectively. None of the placebo recipients formed positive responses. Significantly, the anti-Env response was enhanced about 2-fold after the third dose of the vaccine (p= 1.43e-05) decreasing again at week 48 (p= 3.13e-05

*Anti-vaccinia antibodies.* Anti-vaccinia titers were high after the second immunization. The proportion of responders increased from 91.7% at week 8 to 100% at week 18. Thereafter, it decreased to 77.3% at week 48.

*Neutralizing antibodies.* Neutralizing activity from serum of volunteers was measured at week 48 against HIV-1 primary isolate BX08. After three vaccinations of MVA-B, 8 out of 24 volunteers (33%) were able to neutralize with a titer > 1/90 (that we consider as positive). If we include also patients with a weak positive response (a titer above 1/50) then 13 out of 24 volunteers (54%) show a positive response. None of the placebo recipients gave positive responses. There were no cross-clade neutralizing antibody responses, as no neutralizing activity was detected in serum against virus MN and AC10.

**(ii). Rationale for this study**

This proposal to study a modified pox viral vector is innovative in the following:

1. There are no data of other candidates about the durability at long-term of immunological response to an HIV/AIDS vaccine.
2. It is important to know if a new dose of the same immunogen after long-term could be safe and boost the immune response further.

# II Summary of Trial

24 healthy male and female volunteers in Barcelona and Madrid who are at low risk of HIV infection and entered into the RISVAC02 study and were randomly allocated to receive 3 intramuscular injections of MVA-B at weeks 0, 4 and 16 will receive a boosting dose 4 years thereafter.

Participants will attend one of two clinical centres on at least 5 occasions over 16 weeks. These visits will comprise:

- Screening
- Trial entry and boosting immunisation
- Early follow-up after immunisation
- Follow-up x 2 including the final visit

Participants will have blood and urine collected, and receive 1 immunisation. They will be counselled prior to and following a HIV test, and given health education on prevention of sexually transmitted infections including HIV. They will receive 350 € to reimburse their travelling and inconvenience caused by frequent attendance at hospital.

The two centres are:

- Hospital Clinic, Barcelona and
- Hospital Gregorio Marañón, Madrid

The primary objective is to explore the safety and immunogenicity of MVA-B, and the respective end-points are:

- Safety: grade 3 or above local (pain, rash, swelling), general (fever, chills, headache, nausea, vomiting, malaise, myalgia) and other unsolicited adverse events.
- Immunogenicity: cellular responses assessed using IFN-γ ELISPOT technique.

The data management and analysis will be coordinated by the Hospital Clinic Clinical Trials Unit and the randomisation and serious adverse event reporting number is:

**00 34 932275400 (ext 2815)**

The trial will be overseen by a Trial Coordinating Committee which will include the Principal Investigators from each centre and two independent members including the Chair. A Data and Safety Monitoring Committee will also be appointed to review the design and protocol, but will only meet during the trial if 3 or more participants experience an unexplained, unexpected grade 3 or 4 clinical or laboratory event (confirmed on attendance or repeat testing), not resolved within 72 hours and considered probably or possibly related to vaccine.

Internationally accepted good manufacturing and good clinical practices will be followed. Screening will not commence until the ethical approval local to the centre is obtained and immunisations will not commence until the regulatory approval appropriate to the clinical centre is in place.

# III Allocation of immunisations

The products will be in liquid form and must be stored at –20^o^C, and thawed at room temperature prior to use. The volume is 1ml in 2ml vials. The vials should be gently swirled but not inverted. The immunisations will be given into the non-dominant deltoid muscle.

| **Time in weeks** |  |  | **0** |
| --- | --- | --- | --- |
|  | **Site** | **Number** |  |
| **Vaccine groups** | Barcelona | 12 | ~1x10^8^ pfu/ml MVA-B* |
|  | Madrid | 12 | ~1x10^8^ pfu/ml MVA-B* |

IV Scientific questions to be addressed **and analyses:**

| **Question** | **Groups** | **Primary safety end-points** |
| --- | --- | --- |
| Is the novel regimen safe? | 1 | Grade 3 or above adverse event within 28 days of any vaccination expressed as a proportion with confidence intervals |
|  |  | **Primary immunogenicity end-points:** |
| How immunogenic is the MVA-B against clade B peptides? | 1 | ELISPOT responses following immunisations to pools of B peptides during this trial |

Detailed study schedule in section 2.6

# 1. General plan

## 1.1 Objectives

The primary objective is to explore the safety and immunogenicity of one boost of MVA HIV-B in healthy male and female volunteers at low risk of HIV infection after 4 years of receiving a complete 3 injections schedule.

## 1.2 Design

The design does not include randomisation since this is an open roll-over study of a clinical trial ended 4 years before.

## 1.3 Population.

The study will include 24 healthy male and female volunteers HIV negative and at low risk of HIV infection who were entered into the RisVac02 study and allocated to receive 3 intramuscular injections of MVA-B. The volunteers will receive an additional boost after 4 years.

Selection criteria for RisVac02 study at the entry of the study were:

| Inclusion Criteria |
| --- |

- male or female
- age between 18 and 55 years on the day of screening
- available for follow-up for the duration of the study (52 weeks from screening)
- able to give written informed consent
- at low risk of HIV and willing to remain so for the duration of the study

**low risk of HIV infection defined as:**

- no history of injecting drug use in the previous ten years
- no gonorrhoea or syphilis in the last six months
- no high risk partner (e.g. injecting drug use, HIV positive partner) either currently or within the past six months
- no unprotected anal intercourse in the last six months
- no unprotected vaginal intercourse outside a relationship with a regular known/presumed HIV negative partner in the last six months
- willing to undergo a HIV test
- willing to undergo a genital infection screen
- if heterosexually active female, using an effective method of contraception with partner (combined oral contraceptive pill; injectable contraceptive; IUCD; consistent record with condoms if using these; physiological or anatomical sterility in self or partner) from 14 days prior to the first vaccination until 4 months after the last, and willing to undergo urine pregnancy tests prior to each vaccination
- if heterosexually active male, using an effective method of contraception with their partner from the first day of vaccination until 4 months after the last vaccination

| Exclusion Criteria |
| --- |

- positive for hepatitis B surface antigen, hepatitis C antibody, antibody responses to vaccinia or serology indicating active syphilis requiring treatment
- pregnant or lactating
- clinically relevant abnormality on history or examination including history of grand-mal epilepsy, severe eczema, immunodeficiency or use of immunosuppressives in preceding 3 months
- receipt of live attenuated vaccine within 60 days or other vaccine within 14 days of enrolment
- receipt of blood products or immunoglobin within 4 months of screening
- participation in another trial of a medicinal product, completed less than 30 days prior to enrolment
- history of severe local or general reaction to vaccination defined as

**local**: extensive, indurated redness and swelling involving most of the antero-lateral thigh or the major circumference of the arm, not resolving within 72 hours

**general**: fever >= 39.5^o^C within 48 hours; anaphylaxis; bronchospasm; laryngeal oedema; collapse; convulsions or encephalopathy within 72 hours

- HIV 1/2 positive or indeterminate on screening
- positive for hepatitis B surface antigen, hepatitis C antibody or serology indicating active syphilis requiring treatment
- grade 1 routine laboratory parameters (see section 4.1.4 & appendix 4 for definitions)
- unlikely to comply with protocol

Before entering to RisVac02 boost study, the investigator will verify that all volunteers continue fulfilling the same criteria selection that in the RisVac02 study.

## 1.4 Trial products

**1.4.1 Supply, storage and composition of MVA-B and placebo**

**Impfstoffwerk Dessau-Tornau GmbH (IDT)** is responsible for manufacture and supply of all clinical material according to Good Manufacturing Practice. The presentation is in a liquid form, 1ml in single dose 2ml vials, which should be stored at -20^o^C. The composition is below:

| **Ingredients** | **MVA HIV-B** |
| --- | --- |
| 1. Active substances | |
| Modified Pox virus, strain MVA clade -B (expressing HIV-1 Bx08gp120 and IIIB gagpolnef) | ~ 1 x 10^8^ pfu/ml  -- |
| 2. Excipients | |
| Tris (hydroxymethyl)-amino methane | 0.121 mg/ml |
| Sodium Chloride | 0.818 mg/ml |
| 3. Impurities | |
| Chicken Embryo Fibroblast protein | Traces  --  ≤ 20µg/ml  -- |
| Gentamycin |

**1.4.2 Preparations prior to use**

Prior to use the vials should be thawed at room temperature. When completely thawed the vials should be gently swirled. Care must be taken not to invert the vials.

- - 1. **Labels**

MVA-B vials will be labelled by IDT. The label will contain the information including but not limited to the clinical sites and principal investigators for each centre, the storage details and the name of the supplier for the product.

**1.4.4 Dispensing records and disposal of unused product**

The designated pharmacist will, upon receipt of supplies prior to commencement of the trial, conduct an inventory and complete a receipt. During the trial the pharmacist will be responsible for reviewing the dispensing log.

On the day of immunisation, and with the participant present, the Investigator will complete a prescription with the trial number and date of birth. The data will be entered against the trial number in the dispensing log.

The individual who administers the injection will be responsible for ensuring that the return of the used vials is recorded in the dispensing log at the end of the clinical session.

At the end of the trial all used and unused vials will be checked against the inventory by staff from Hospital Clinic CTU before returning to the supplier or disposal on site according to local pharmacy guidelines and applicable regulations. Documentation of disposal will be provided to Hospital Clinic CTU and the supplier.

During the trial, product accountability will be monitored by the prescriptions, the dispensing log, the returns, the trial register and data collected on the case report forms.

## 1.5 End-points

**1.5.1 Primary**

The primary endpoints are safety and immunogenicity as defined below.

The primary safety parameters will be graded according to appendix 4, and are:

- Grade 3 or above local adverse event (pain, cutaneous reactions including induration)
- Grade 3 or above systemic adverse event (temperature, chills, headache, nausea, vomiting, malaise, and myalgia)
- Grade 3 or above other clinical or laboratory adverse event confirmed at examination or on repeat testing respectively
- Any event attributable to vaccine leading to discontinuation of the immunisation regimen

Data on local and systemic events listed above will be solicited with specific questions for a minimum of 7 days following the immunisation. Data on other clinical and laboratory events will be collected with an open question at each visit and through routine scheduled investigations respectively.

The primary immunogenicity parameters will be quantitative or present/absent, and are:

- cellular responses - CD8/CD4+ T cell responses (ELISPOT) at week 2, 4 and 12 following the immunisations
  - 1. **Secondary**

Secondary safety and immunogenicity end-point information will be collected on all participants on the following:

- all grade 1 and 2 adverse events within 28 days of vaccination
- antibody responses
- binding titration to the construct MVAB
- binding titration to and neutralisation of vaccinia
- cellular responses
- CD8/CD4+ T cell responses (ELISPOT) at week 0
- intracellular cytokine analysis at week 0, 2, 4 and 12

# 2 Schedule of Visits

## 2.1 Recruitment

Healthy volunteers recruited in RISVAC02 who received vaccine will be asked to participate in the study.

If they are interested and willing to participate, they will be invited to attend for screening.

## 2.2 Screening

At this visit, which must take place within 42 days of the randomisation visit, the study will be discussed in detail. Any questions about the study will be answered. If volunteers are still willing and interested they will be asked to sign part 1 of the informed consent form (appendix 2).

To ensure informed consent, subjects will go through the following processes in detail with a member of the study team

1. Pre-HIV test counselling
2. Safe sex counselling
3. That it is unknown whether or not the active study vaccine will protect against HIV infection
4. That following immunisation they may develop antibodies that will produce a positive reaction in a routine HIV test, but that provisions have been made to distinguish between a post vaccination response and HIV infection during and after the trial
5. The level of care that will be made available to them should they be found to be HIV infected at any time during their participation in the study, including the screening period.
6. That they, or their partner should continue to use a reliable form of contraception for 14 days prior to the immunisation period and for 4 months afterwards
7. That they should continue to use condoms with sexual partners whose HIV status is not known
8. That they may be subject to social risk if they develop HIV antibodies, or by revealing their participation in the study

After informed consent has been collected, assessments and procedures will be undertaken according to the schedule 2.6, including a physical examination and collection of specimens for laboratory investigations. Details of these are given in sections 3 and 4. The investigator will enter the data collected onto the case record form.

## 2.3 Trial Entry

The results of the screening investigations will be reviewed and volunteers who are still eligible and willing will be asked to complete part 2 of the informed consent. This will include asking for consent to undertake follow-up by direct contact. The information about this project and consent to participate will be separate to the main consent and will not be compulsory (appendix 2b). They will be strongly advised to inform their current general practitioner of their participation but this will not be a pre-requisite to enrolment. Assessments and procedures will be undertaken according to the schedule 2.6, and data entered on the case record form.

Trial product will be dispensed as outlined in 1.4.3.

- - 1. **Immunisation**

Immunisations will be given at 0 week into the deltoid muscle of the non-dominant arm, by a physician or nurse. The overlying skin will be stretched flat prior to insertion of the needle. To ensure that the needle reaches the muscle, and that product does not seep into the surrounding subcutaneous tissues, a decision about the needle length required will be made individually for each participant. For ten minutes after immunisation, the participant will remain in the clinical room, after which the injection site will be inspected, and a plaster will be placed over the site. These precautions are to exclude the possibility of MVA B transmission to non-vaccinees, although in reality there is no risk of viral shedding.

The immunisations will occur in an outpatient setting, and participants will be closely observed for one hour after immunisation, at which point vital signs (pulse, blood pressure and respiratory rate) will be recorded on the case record form, as will any local reactions (after removal of the plaster) and systemic events.

The plasters, needles and syringes used for all immunisations will be autoclaved or placed in hypochlorite solution at the end of the clinical session.

**2.3.2 Follow-up in the days following immunisation**

A diary card will be given, with instructions and a full verbal explanation, for participants to record local and systemic adverse events following immunisation.

Participants will be observed on the day of immunisation. Contact will be maintained by the clinical team, using a method chosen by the participant, as indicated by the evolution of adverse events, up to resolution of solicited local and systemic events. Additional visits may be recommended at the discretion of the clinical and principal investigators, if clinically indicated or in order to clarify observations.

## 2.4 Follow-up visits

Assessments and procedures will be performed according to schedule 2.6.

## 2.5 Final visit

Assessments will be undertaken according to schedule 2.6. The importance of follow-up will be emphasised at this visit and more than one method of contacting participants collected.

**2.5.1. Reimbursement**

Regular payments will be made to all participants to cover their travel expenses and any inconvenience caused. On completion of the study participants will have received a total of 350 €. Payment will be made to those who do not complete the study according the number of visits that they have completed (70 € per visit). Those who have to attend for additional visits will be reimbursed accordingly. During the study participants will receive latex condoms free of charge. If any medication is required as a result of the study, this will also be provided free of charge.

**2.5.2. In the event of discontinuation**

It is possible that a participant may develop a condition, which regardless of the relationship to study product, may cause the Investigator to discontinue them from further immunisations. Follow-up should carry on whenever possible up to the time of the final visit, but at least until resolution or stabilisation of the condition. The date that the participant is discontinued from further immunisations and the reason will be recorded in the case record form.

## 2.6 Study schedule

((X) indicates that a specimen will only be collected if indicated by history)

| Visit Number | 1 | 2 | 3 | 4 | 5 | 6 | Vol  blood in ml |
| --- | --- | --- | --- | --- | --- | --- | --- |
| Nominal week (+/-week) | Up to -6 | 0 (+/-2) | 1 | 2 (+/-3dys) | 4 (+/-1) | 12 (+/-2) |
| Immunisation |  | x |  |  |  |  |
| Eligibility | X |  |  |  |  |  |  |
| History & exam^a^ | X |  |  |  |  |  |  |
| Genital infection screen^b,c^ | X |  |  |  |  |  | 12 |
| HIV risk screen | X |  |  |  |  |  |  |
| HIV Ab test^c^ | X | X |  |  |  | X | 6 |
| Safe sex counselling | X | X |  |  |  | X |  |
|  |  |  |  |  |  |  |  |
| Adverse event assessment |  | X | X |  | X | X |  |
| Haematology | X | X |  |  | X | X | 4 |
| Chem pathology | X | X |  |  | X | X | 5 |
| CD4 number & percentage  urine | X | X |  |  |  |  | 4 |
| IgG, IgA, IgM | X |  |  |  |  |  |  |
| Urinalysis | X | X |  |  | X |  |  |
| Pregnancy test, if female^c^ |  | X |  |  | (X) |  |  |
|  |  |  |  |  |  |  |  |
| Store for HLA if needed | X |  |  |  |  |  | 5 |
|  |  |  |  |  |  |  |  |
| Antibody responses |  | X |  | X | X | X | 10 |
| ELISPOT responses |  | X |  | X | X | X | 75 |
| Flow cytometry |  | X |  | x | x | x |
| Cell storage |  | X |  | X | X | X |
|  |  |  |  |  |  |  |  |
| Diary card |  |  |  |  |  |  |  |
| Contact by trial staff |  |  |  |  |  |  |  |
|  |  |  |  |  |  |  |  |
| Payment |  |  |  |  | x | X |  |

^a^including weight in kg, height in cm and arm circumference in cm

^b^including serology for syphilis, hepatitis B and C at screening. Genital swabs (or urine) for Neisseria gonorrhoea, chlamydia trachomatis and trichomonas vaginalis will be collected if indicated according to clinical standardised operating procedures (SOPs).

^c^ additional HIV/STI and pregnancy tests will be performed if indicated by a change in risk status or menstrual history respectively

# 3. Procedures

## 3.1 Blood and urine collection

Blood will be collected using a sterile needle, usually from the ante-cubital fossa, according to the schedule and transported to the appropriate laboratories. Urine will be collected into a sterile container as indicated on the schedule and either transported to the appropriate laboratory or tested by a member of the clinical team according to trial specific standardised operating procedures.

**3.2 Clinical history and examination**

A past medical history will be collected using the screening proforma at the screening visit, including details of any previous reaction to vaccination, history of epileptic fit, exposure to vaccinia, and contraceptive practices. The general examination will include weight (kg), height (cm) and arm circumference (see 4.1.2), blood pressure, inspection of the skin to exclude severe eczema and check for the presence of a vaccinia scar, respiratory, cardio-vascular and abdominal systems examination. An assessment of cervical and axillary lymph nodes will also be undertaken. The examination results and history of exposure to vaccinia will be recorded on the case record form.

## 3.3 Genital infection screen

The following will be collected in all participants

- serology for syphilis
- serology for markers of hepatitis B surface antigen or hepatitis C antibody

## 3.4 Pre-HIV test screen and counselling

Study personnel will assess volunteers for past and current risk of HIV infection using the screening proforma and counsel them prior to collecting blood for a HIV test. The counselling process will ensure that volunteers have sufficient knowledge about HIV infection to understand what the test is for, the implications of a positive, negative and equivocal result and the standard of care available for HIV infection locally. They will also be informed how and when they will receive the result, according to the local policy in each clinical centre.

## 3.5 Safe sex counselling and condom provision

Participants will be counselled by study personnel about the importance of condoms at screening and reminded on the day of immunisation. Hypoallergenic condoms will be provided free of charge to participants throughout the trial.

## 3.6 HIV related issues

Please see section 6 for details of the procedures which may be required in the event of:

1. a request for a HIV test
2. HIV infection
3. Social discrimination as a result of post-vaccine response

## 3.7 Discontinuation procedures (including withdrawal)

Participants may withdraw at any time if they wish to do so, for any reason. The date of withdrawal and reason for doing so should be recorded in the final visit case record form.

The discontinuation and reason should be recorded on the case record form. In case of discontinuation due to adverse event, the adverse event should be recorded on the adverse event case report form. Trial visits should carry on at least until resolution or stabilisation of the event, but ideally up to the last visit in the schedule, provided the participant is willing. The frequency of visits and laboratory investigations may be reduced on consultation with the Principal Investigator or Trial Management Group (see section 7).

# 4. Assessments

## 4.1 Safety assessments

**4.1.1 Local adverse events**

Pain in the muscle injected will be graded by the participant according to the criteria in appendix 4 as mild (1) moderate (2), severe (3) or extreme (4) and recorded in the appropriate case record form or once the participant has left clinic, in the diary card.

Redness will be recorded as the maximum diameter expressed as a proportion of the arm circumference and graded according to appendix 4. Arm circumference will be measured at screening at the point one third of the way down from the acromio-clavicular joint towards the elbow joint of the non-dominant arm. Participants will be asked to record the maximum diameter in the diary card, as well as the presence of any itching or other discomfort and any medication taken for relief of symptoms.

Blistering (vesiculation) or ulceration will be graded according to size and depth, and induration according to size (see appendix 4).

**4.1.2 Systemic adverse events**

Temperature will be measured prior to immunisation and one hour later by study personnel, and graded according to appendix 4. Participants will be given a thermometer to record their temperature in the diary card on the evening of immunisation, and daily thereafter for 7 days, and if still raised, they will be advised to continue to monitor their temperature daily until it returns to normal. The temperature observed by the clinical team will be recorded on the case record forms for visits.

Chills, headache, nausea, vomiting, malaise and myalgia will be graded by the participants according to appendix 4, recorded in the diary card on the evening following immunisation and daily for 7 days, or until resolution of symptoms whichever is longer. On days where a visit coincides with the diary card, the clinical staff will collect the information directly and record it on the case record form, and this will take precedence over diary card entries in the analysis.

**4.1.3 Other adverse events**

These will be recorded as reported following an open question to participants, with the dates of commencement and resolution and any medication required. They will be graded according to the general principles outlined in appendix 4. Social harm will also be recorded as an adverse event, graded according to the general guidelines.

**4.1.4 Routine laboratory and urinary parameters**

The following safety assessments will be undertaken in laboratories local to the clinical centre according to standard procedures subject to quality control:

- haematology: haemoglobin, white cell and platelet count, neutrophils

and lymphocytes

- chemical pathology: liver function tests (AST/ALT, alkaline phosphatase,

bilirubin), creatinine, glucose

- immunology: CD4 number and percentage

immunoglobulins A, G and M

The following assessment will be undertaken on a specimen of urine, conducted by a member of the study staff according to trial specific standardised operating procedures:

- urinalysis: normal/abnormal dipstick test conducted by clinical staff

## 4.2 Immunogenicity assessments

**4.2.1 Antibody responses**

Antibodies to the construct will be assessed using ELISA according to standardised operating procedures in a single research laboratory. Validation of the results will be undertaken including analysis of plates for all participants at a single timepoint (4 weeks after the immunisation) and all samples from a single participant selected at random.

Antibodies to vaccinia virus in serum from  healthy volunteers, before and after vaccination, will also be assessed in a single research laboratory by ELISA, using cell extracts from monkey BSC-40 cells infected (1 pfu/cell)  with vaccinia virus (strain WR) for 24 h, and by neutralization titer of the mature virus form (MV) of vaccinia virus (WR). Appropriate international standards will be used, like  the National Institute of Biological Standards and Control (reference NIBSC 63/024).

**4.2.2 Cellular responses**

##### Primary cellular responses will be assessed using the ELISPOT assay on frozen specimens according to standardised operating procedures (SOPs). These procedures will be validated prior to the trial. The ELISPOT reaction per well will be measured in an automated system, according to operating procedures that define the validity of the assay. The frozen specimens can be assessed at a single timepoint (2/4 weeks after the immunisation), and for a single participant as part of the validation procedures. Quality control and exchange of specimens between the laboratories are part of the SOPs.

CD4 and CD8 T cell responses will be assessed by flow cytometry on frozen specimens according to standardised procedures. T-cell responses will be evaluated by the percentages of cells producing IL-2 and/or IFN-γ within CD4 and CD8 T cell populations after 18 hours of ex-vivo stimulation of PBMCs with HIV-1 peptide pools.

## 4.3 Other assessments

**4.3.1 HIV antibody test**

Samples will be tested in the laboratories local to the clinical centre using ELISA, according to standard procedures subject to quality control.

**4.3.2 Pregnancy test**

A pregnancy test will be performed by analysis of a urine sample for Human Chorionic Gonadotrophin (HCG) collected from female participants at screening and on the day of immunisation. The analysis will be conducted by a member of the study team according to trial specific standardised operating procedures.

**4.3.3 Genital infection**

The assays will be conducted in laboratories local to the clinical centres, according to standard procedures subject to quality control.

**4.3.4 Following adverse event**

Other assessments may be performed as clinically indicated due to an adverse event.

- - 1. **Concomitant medication**

Participants will be asked about medication taken at each visit, and this will be recorded in the case record form. Thereafter, they will be asked about medication only in relation to adverse events reported. It is expected that the name of the drug, indication for use, dose, frequency, start and stop dates will be available for prescription-only medication, either from the participant or from the prescribing physician. For medication available over the counter, the maximum information available on questioning the participant will be recorded.

# 5. Adverse events

## 5.1 Definitions

An adverse event is any adverse experience occurring during the course of the study including the screening period.

Criteria for grading clinical and laboratory events are listed in appendix 4.

A severe adverse event is one **graded 3 or 4** by criteria in appendix 4. Some, but not all grade 3 and 4 adverse events will be “serious” by ICH GCP criteria below.

**5.1.1 Serious Adverse Events (SAEs)**

An adverse event is considered to be a “serious adverse event” by ICH Good Clinical Practice (ICH GCP) criteria if it results in the following:

- death,
- a threat to life,
- requires in-patient hospitalisation or prolongs existing hospitalisation (hospitalisation for elective treatment of a pre-existing condition is not included),
- results in persistent or significant disability or incapacity,
- is a congenital anomaly (i.e., the outcome of pregnancy involving a participant), or
- is any other important medical condition*.

*Examples of conditions regarded as “any other important medical condition” include allergic bronchospasm requiring intensive emergency treatment, seizures or blood dyscrasias which did not result in hospitalisation or development of drug dependency.

## 5.2 Relationship to study product

This can be classified as:

**Unrelated** adverse events that can be clearly explained by extraneous causes and for which there is no plausible association with study product, or adverse events for which there is no temporal relationship

**Unlikely to be** adverse events that may be temporally linked, but which are much more likely to be due to other causes than study product and which do not get worse with continuing use of product

**Possibly** adverse events that could equally well be explained by study product or other causes, which are usually temporally linked and may improve when not using study product but do not reappear when using study product

**Probably** adverse events that are temporally linked and for which the study product is more likely to be the explanation than other causes, which may improve when not using study product

**Definitely** adverse events that are temporally linked and for which the study product is the most likely explanation, which disappear or decrease when not using study product and reappear when using study product

## 5.3 Reporting adverse events

Adverse events should be recorded on the appropriate case record form.

Any grade 3 or 4 adverse event (see appendix 4) or any event resulting in discontinuation of the vaccination schedule should be reported within 2 working days to the Hospital Clinic CTU of the decision to discontinue.

SAEs that are considered possibly, probably or definitely related should be reported to the Hospital Clinic CTU the same working day that the Clinical Investigator becomes aware of the event fulfilling the above criteria. This can be done by telephone or fax. The minimum criteria required in reporting a SAE are the participant identifiers (trial number/ date of birth/initials), reporting source (name of Investigator), and why the adverse event is identifiable as serious, and relationship to study product.

**The adverse event reporting telephone number is 00 34 932275400 (ext 2815)**

**and fax is 00 34 932279877**

Staff at the Hospital Clinic CTU will confirm that the event qualifies as a Suspected Unexpected Adverse Drug Reaction (SUSAR) and arrange for urgent review of the case to take place within 2 working days. Those involved in this review will include the site Principal Investigator, the medical expert at CTU, and a clinician who is an independent member of the Trial Coordinating or Data Safety Monitoring Committee. The report will be prepared by the CTU Medical Expert and filed with the appropriate regulatory authorities, and with the Hospital Clínic, within the timelines required by national legislation. The CTU Medical Expert will inform the full Trial Coordinating and Data and Safety Monitoring Committees. The site Principal Investigator is responsible for notifying their Local Research Ethics Committee.

## 5.4 Clinical management

Events will be managed by the clinical trial team who will assess and treat the event as appropriate, including referral to an independent physician and/or the participant’s General Practitioner if required. There will be clinical operating procedures in place for the management of abnormalities detected following urinalysis or routine laboratory tests.

# 6 Management of HIV issues during and following the trial

## 6.1 HIV testing

Only volunteers with a negative HIV ELISA result will be enrolled. It is possible that participants may develop antibodies and test ‘positive’ in routine HIV ELISA assays subsequent to immunisation. Designated laboratories local to the clinical centres, will conduct any additional tests to distinguish between infection and a post-immunisation response required either for clinical management, or at the request of a participant. Entry and week 12 samples will be collected, separated, stored and batched for transport to these laboratories, and tested at the end of the trial. Week 12 specimens will be tested in real-time so that participants can be informed of the result before the end of the trial and a plan to recall and retest them made should this be necessary. In the event of ongoing post-immunisation positive ELISA, the participants will be invited to reattend annually until such time as this response has disappeared, and provided with an explanatory identity card in the interim.

**6.1.1 Verification of HIV status of participants**

If certification is required at the request of the participant, this can be provided by the trial team after testing at the local laboratory.

Results will always be given at an interview with a member of the study team unless the participant requests an independent physician, in which case this will be arranged.

If a specimen from a participant suggests that they are HIV infected, a second specimen will be collected and retested.

## 6.2 HIV infection

In the unexpected circumstances that a participant in the trial acquires HIV infection, they will be managed in the following way:

**6.2.1 Referral for clinical care**

Participants will be referred initially to a specialist physician at each clinical centre for a full discussion of the clinical aspects of HIV infection. Further investigations will be undertaken as necessary. Should the participant prefer to be managed at a hospital closer to their home, or by their General Practitioner, this will be arranged.

**6.2.2 Referral for counselling:**

This will be arranged by the specialist physician, to a counsellor at their clinical centre. The counselling process will assist the participant in the following issues:

- psychological and social implications of HIV infection
- who to inform and what to say
- implications for sexual partners
- avoidance of risk to others in future

**6.2.3 Informing the General Practitioner**

The participant will be encouraged to do this, but the decision will remain at the discretion of the individual.

**6.2.4 Immunological follow-up**

Follow-up of HIV infected individuals who have received study vaccine products will be determined by the Trial Management Group. The intensity of assessments will be dependent on their clinical progress, including changes in surrogate markers such as viral load and CD4 count.

## 6.3 Social discrimination as a result of a post-vaccine response

The aim is to minimise the possibility of social discrimination in participants who develop a positive HIV-ELISA test by providing HIV testing and certification for participants as required, outlined above, both during and after the trial. In addition, an identification card stating that the individual has participated in a vaccine trial, giving a contact number in case of medical emergency will be provided.

In the unlikely event that a participant suffers social discrimination as a result of a post-vaccination response, the clinical investigators will assist the participant.

# 7. Management of the trial

## 7.1 Data management at the Clinical Centres

Hospital Clinic and Hospital Gregorio Marañón will be responsible for:

- Entering relevant information (see section 7.5) in the clinical notes, and holding a record for each participant which includes the bottom copy of the CRF with any changes made signed and dated
- The accurate completion of the case record forms

Data will be recorded directly onto the case report forms, which will provide the majority of source data for the trial. There will be some additional source data in the clinical notes, such as medical history related to eligibility, dates visits including immunisation and details of clinical management (adverse events and concomitant medication).

Duplicate case record forms (CRFs) will be supplied by the Hospital Clinic CTU and the top copy returned to CTU for data entry after completion. In the event of an abnormality, an indication should be given whether or not action was taken, the date of review and the signature of the clinician reviewing the result.

Changes to the CRF should be signed and dated, including changes made before the form is returned to the Hospital Clinic CTU.

CRFs and clinical notes should be kept in a secure location for 2 years after the last approval of a marketing application or until 2 years have elapsed since formal discontinuation of product development.

## 7.2 Data management in the immunology laboratories

Standardised operating procedures will be followed in all laboratories to ensure the quality of the data. Data will be stored electronically in an agreed format and datafiles transferred to CTU for the main analysis.

## 7.3 Data management at the Hospital Clinic CTU

Hospital Clinic CTU will be responsible for:

- Design of the case record forms in collaboration with the Investigators
- Monitoring the trial according to Hospital Clinic GCP guidelines (derived from ICH guidelines) including monitoring vaccine accountability, and dispatch and arrival of immunological specimens
- Preparation of reports to assist the monitoring
- Holding a record for each participant which contains the original top copy of the CRF and documentation detailing all the changes made subsequent to monitoring visits, queries raised and how they were addressed
- Coordination of the committee and group meetings (section 7.6 and 7.8) in collaboration with the Investigators
- Coordination of end-point committee meetings, if required, to review the grading of adverse events and their relationship to vaccine product and ELIPSPOT results
- Preparation of analysis files from the database prior to analyses

A printout of the entered data will be generated for each participant CRF in order to cross check the trial database against the CRFs to validate data entry.

The data manager or their deputy will review adverse events, as they arise. Queries raised will be directed to the investigators at Hospital Clínic or Hospital Gregorio Marañón by letter, fax, email or at a monitoring visit.

Prior to analysis, the safety data will be checked, adverse events validated and data extracted in order for the trial statisticians to run the analysis and prepare the tables.

## 7.4 Monitoring by HOSPITAL CLÍNIC CTU

Staff from the Hospital Clinic CTU will visit the clinical centres to validate trial data held on the database against the clinical records, and monitor the centre’s adherence to GCP and the trial protocol. The Clinical Investigators and participants, by giving consent, agree that the Hospital Clinic CTU may consult and/or copy source records (clinical notes and laboratory values) in order to do this. Such information will be treated as strictly confidential and will in no circumstances be made publicly available. The monitoring will adhere to Hospital Clinic Good Clinical Practice guidelines (derived from ICH guidelines). The following data should be verifiable from source documents:

- documentation of any existing conditions or past conditions relevant to eligibility
- signed consent
- dates of visits including dates of immunisations
- a sample of reported laboratory results
- a description and measurements of cutaneous reactions to immunisation
- grade 3 or 4 adverse events and any events leading to discontinuation of the immunisation schedule
- concomitant prescribed medication

Vaccine returns will also be monitored at visits to the clinical sites.

## 7.5 Data Ownership

The data generated in this study will be the property the Investigators and will be held on their behalf by Hospital Clinic CTU.

## 7.6 Trial Coordinating Committee (TCC)

The supervision of the trial will be the responsibility of the Trial Coordinating Committee (TCC). The committee will have a chair, Professor José M Gatell, and the members will include Dr. Felipe García, Dr. Juan Carlos López Bernaldo de Quirós and one further independent member. This committee will be responsible for final decisions about grade of adverse events and relationship to study vaccine, and the independent members will have an additional casting vote should this be required. Notes of meetings will be kept.

The trial may be terminated by this Committee for any reason, including on the recommendation of the DSMC.

- - 1. **Trial Management Group (TMG)**

This group will oversee the day to day running of the trial and the members will be primarily the clinical and data management teams. The immunologists will join if there are relevant items on the agenda. Notes will be taken and will form the basis of the progress report to the Trial Coordinating Committee.

## 7.7 Data and Safety Monitoring Committee (DSMC)

A DSMC will be invited to oversee this trial. No member of the Trial Management Group, Trial Coordinating Committee, or any Clinical Investigator responsible for the clinical care of trial participants may be a member of the DSMC.

The DSMC will review the design of the trial prior to commencement. The DSMC will not meet unless there are indications for an interim review (see below).

# 8 Statistical considerations

## 8.1 Sample size

It is not the remit of this study to recruit a sufficient number of participants to be statistically confident about the result.

This is an exploratory study, and the immunological analyses will be descriptive.

## 8.2 Analysis

All safety end-points will be graded by the Clinical Investigators and reviewed by the Trial Management Group. Any queries about grade and relationship to study product that cannot be resolved will be referred to the Trial Coordinating Committee for a final decision.

All clinical event and routine laboratory data will be included in the safety analysis. Tables including all events occurring within 28 days of immunisation, and limited to those possibly or probably related to study product will be prepared.

The immunological end-points will be considered as present or absent for the main analysis. There will be an immunological end-point committee, which will undertake a of the ELISPOT assays and attempt to quantify each result.

Figures/tables will be prepared including all participants’ results, and limited to those participants who completed the full schedule.

# 9 Confidentiality, ethics and responsibilities, including indemnity

Full medical confidentiality will be preserved.

The study will be conducted according to the Hospital Clinic GCP guidelines (based on ICH guidelines) and the Declaration of Helsinki (version 1996), and it is the responsibility of the Clinical Investigators and the staff at the Hospital Clinic CTU to abide by this protocol.

The Principal and Clinical Investigators are responsible for obtaining the appropriate Local Research Ethics Committee (LREC) approval for the study protocol, the subject information sheet and the consent form. The Principal and Clinical Investigators are responsible for informing the LRECs of any SAEs as required, and submitting annual reports as required.

Regulatory submissions will be made in Spain and these will be coordinated by Hospital Clinic, who will also provide the Investigator Brochures for the products. Regulatory approval must be provided before study materials will be shipped to the clinical centres. IDT is responsible for the supply and labelling of MVA B.

CTU will be responsible for all aspects of data management including monitoring of the clinical sites, and the analysis. Staff will also be responsible for coordinating the response to any SAEs that arise during the course of the trial and reporting these if indicated to the regulatory authorities and to Hospital Clínic in the appropriate time-frames.

Fundació Clínic will act as Sponsor for the trial, and will coordinate the necessary clinical trial agreement delineating the above responsibilities and the liability for events occurring as a result of participating on the trial. This agreement must be signed by all parties involved before materials are shipped to the relevant clinical centre. Indemnity will be ensured by the following parties:

- The Principal Investigators will ensure that all clinical staff engaged in the study are covered for negligent harm to a participant, either through a personal or hospital insurance scheme
- Hospital Clínic agrees to operate in good faith in accordance to the European Union Clinical Trials Directive (2001/20/EC) if an adverse event is thought to be probably or possibly related to MVA B.
- Infectious Diseases Unit, Hospital Clinic will be responsible for ensuring that a policy is in place to cover ‘no fault compensation’ for participants on the trial who suffer an adverse event that is attributable to participation in the trial but which is not related to the manufacturing fault of the product or due to negligent harm caused by the clinical staff.

# 10 Publication

It is intended that the results of this study will be published in an appropriate peer-reviewed journal. The Trial Coordinating Committee will have 30 days to comment on any manuscript. No other publications, whether in writing or verbally, will be made before the definitive manuscript has been agreed and accepted for publication, without the prior approval of this committee.

# 11 References:

1. Grant RM, Lama JR, Anderson PL, McMahan V, Liu AY, Vargas L*, et al.* Preexposure Chemoprophylaxis for HIV Prevention in Men Who Have Sex with Men. *N Engl J Med* 2010; **363(27)**:2587-2599.

2. Abdool KQ, Abdool Karim SS, Frohlich JA, Grobler AC, Baxter C, Mansoor LE*, et al.* Effectiveness and safety of tenofovir gel, an antiretroviral microbicide, for the prevention of HIV infection in women. *Science* 2010; **329(5996)**:1168-1174.

3. Rerks-Ngarm S, Pitisuttithum P, Nitayaphan S, Kaewkungwal J, Chiu J, Paris R*, et al.* Vaccination with ALVAC and AIDSVAX to prevent HIV-1 infection in Thailand. *N Engl J Med* 2009; **361(23)**:2209-2220.

4. Buchbinder SP, Mehrotra DV, Duerr A, Fitzgerald DW, Mogg R, Li D*, et al.* Efficacy assessment of a cell-mediated immunity HIV-1 vaccine (the Step Study): a double-blind, randomised, placebo-controlled, test-of-concept trial. *Lancet* 2008; **372(9653)**:1881-1893.

5. McElrath MJ, De Rosa SC, Moodie Z, Dubey S, Kierstead L, Janes H*, et al.* HIV-1 vaccine-induced immunity in the test-of-concept Step Study: a case-cohort analysis. *Lancet* 2008; **372(9653)**:1894-1905.

6. Pantaleo G, Esteban M, Jacobs B, Tartaglia J. Poxvirus vector-based HIV vaccines. *Curr Opin HIV AIDS* 2010; **5(5)**:391-396.

7. Stickl H, Hochstein-Mintzel V, Mayr A, Huber HC, Schafer H, Holzner A. [MVA vaccination against smallpox: clinical tests with an attenuated live vaccinia virus strain (MVA) (author's transl)]. *Dtsch Med Wochenschr* 1974; **99(47)**:2386-2392.

8. Antoine G, Scheiflinger F, Dorner F, Falkner FG. The complete genomic sequence of the modified vaccinia Ankara strain: comparison with other orthopoxviruses. *Virology* 1998; **244(2)**:365-396.

9. Stickl HA. Smallpox vaccination and its consequences: first experiences with the highly attenuated smallpox vaccine "MVA". *Prev Med* 1974; **3(1)**:97-101.

10. Mwau M, Cebere I, Sutton J, Chikoti P, Winstone N, Wee EG*, et al.* A human immunodeficiency virus 1 (HIV-1) clade A vaccine in clinical trials: stimulation of HIV-specific T-cell responses by DNA and recombinant modified vaccinia virus Ankara (MVA) vaccines in humans. *J Gen Virol* 2004; **85(Pt 4)**:911-919.

11. Cebere I, Dorrell L, McShane H, Simmons A, McCormack S, Schmidt C*, et al.* Phase I clinical trial safety of DNA- and modified virus Ankara-vectored human immunodeficiency virus type 1 (HIV-1) vaccines administered alone and in a prime-boost regime to healthy HIV-1-uninfected volunteers. *Vaccine* 2006; **24(4)**:417-425.

12. Goonetilleke N, Moore S, Dally L, Winstone N, Cebere I, Mahmoud A*, et al.* Induction of multifunctional human immunodeficiency virus type 1 (HIV-1)-specific T cells capable of proliferation in healthy subjects by using a prime-boost regimen of DNA- and modified vaccinia virus Ankara-vectored vaccines expressing HIV-1 Gag coupled to CD8+ T-cell epitopes. *J Virol* 2006; **80(10)**:4717-4728.

13. Keefer MC, Frey SE, Elizaga M, Metch B, De Rosa SC, Barroso PF*, et al.* A phase I trial of preventive HIV vaccination with heterologous poxviral-vectors containing matching HIV-1 inserts in healthy HIV-uninfected subjects. *Vaccine* 2011; **29(10)**:1948-1958.

14. Ramanathan VD, Kumar M, Mahalingam J, Sathyamoorthy P, Narayanan PR, Solomon S*, et al.* A Phase 1 study to evaluate the safety and immunogenicity of a recombinant HIV type 1 subtype C-modified vaccinia Ankara virus vaccine candidate in Indian volunteers. *AIDS Res Hum Retroviruses* 2009; **25(11)**:1107-1116.

15. Vasan S, Schlesinger SJ, Chen Z, Hurley A, Lombardo A, Than S*, et al.* Phase 1 safety and immunogenicity evaluation of ADMVA, a multigenic, modified vaccinia Ankara-HIV-1 B'/C candidate vaccine. *PLoS One* 2010; **5(1)**:e8816.

16. Currier JR, Ngauy V, de Souza MS, Ratto-Kim S, Cox JH, Polonis VR*, et al.* Phase I safety and immunogenicity evaluation of MVA-CMDR, a multigenic, recombinant modified vaccinia Ankara-HIV-1 vaccine candidate. *PLoS One* 2010; **5(11)**:e13983.

17. Russell ND, Graham BS, Keefer MC, McElrath MJ, Self SG, Weinhold KJ*, et al.* Phase 2 study of an HIV-1 canarypox vaccine (vCP1452) alone and in combination with rgp120: negative results fail to trigger a phase 3 correlates trial. *J Acquir Immune Defic Syndr* 2007; **44(2)**:203-212.

18. McCormack S, Stohr W, Barber T, Bart PA, Harari A, Moog C*, et al.* EV02: a Phase I trial to compare the safety and immunogenicity of HIV DNA-C prime-NYVAC-C boost to NYVAC-C alone. *Vaccine* 2008; **26(25)**:3162-3174.

19. Bart PA, Goodall R, Barber T, Harari A, Guimaraes-Walker A, Khonkarly M*, et al.* EV01: a phase I trial in healthy HIV negative volunteers to evaluate a clade C HIV vaccine, NYVAC-C undertaken by the EuroVacc Consortium. *Vaccine* 2008; **26(25)**:3153-3161.

20. Sandstrom E, Nilsson C, Hejdeman B, Brave A, Bratt G, Robb M*, et al.* Broad immunogenicity of a multigene, multiclade HIV-1 DNA vaccine boosted with heterologous HIV-1 recombinant modified vaccinia virus Ankara. *J Infect Dis* 2008; **198(10)**:1482-1490.

21. Harari A, Bart PA, Stohr W, Tapia G, Garcia M, Medjitna-Rais E*, et al.* An HIV-1 clade C DNA prime, NYVAC boost vaccine regimen induces reliable, polyfunctional, and long-lasting T cell responses. *J Exp Med* 2008; **205(1)**:63-77.

22. Stickl H, Hochstein-Mintzel V, Mayr A, Huber HC, Schafer H, Holzner A. [MVA vaccination against smallpox: clinical tests with an attenuated live vaccinia virus strain (MVA) (author's transl)]. *Dtsch Med Wochenschr* 1974; **99(47)**:2386-2392.

23. Ramirez JC, Gherardi MM, Esteban M. Biology of attenuated modified vaccinia virus Ankara recombinant vector in mice: virus fate and activation of B- and T-cell immune responses in comparison with the Western Reserve strain and advantages as a vaccine. *J Virol* 2000; **74(2)**:923-933.

24. Barouch DH, Santra S, Kuroda MJ, Schmitz JE, Plishka R, Buckler-White A*, et al.* Reduction of simian-human immunodeficiency virus 89.6P viremia in rhesus monkeys by recombinant modified vaccinia virus Ankara vaccination. *J Virol* 2001; **75(11)**:5151-5158.

25. Ourmanov I, Brown CR, Moss B, Carroll M, Wyatt L, Pletneva L*, et al.* Comparative efficacy of recombinant modified vaccinia virus Ankara expressing simian immunodeficiency virus (SIV) Gag-Pol and/or Env in macaques challenged with pathogenic SIV. *J Virol* 2000; **74(6)**:2740-2751.

26. Amara RR, Villinger F, STAPRANS SI, Altman JD, Montefiori DC, Kozyr NL*, et al.* Different patterns of immune responses but similar control of a simian-human immunodeficiency virus 89.6P mucosal challenge by modified vaccinia virus Ankara (MVA) and DNA/MVA vaccines. *J Virol* 2002; **76(15)**:7625-7631.

27. Cosma A, Nagaraj R, Buhler S, Hinkula J, Busch DH, Sutter G*, et al.* Therapeutic vaccination with MVA-HIV-1 nef elicits Nef-specific T-helper cell responses in chronically HIV-1 infected individuals. *Vaccine* 2003; **22(1)**:21-29.

28. Mwau M, Cebere I, Sutton J, Chikoti P, Winstone N, Wee EG*, et al.* A human immunodeficiency virus 1 (HIV-1) clade A vaccine in clinical trials: stimulation of HIV-specific T-cell responses by DNA and recombinant modified vaccinia virus Ankara (MVA) vaccines in humans. *J Gen Virol* 2004; **85(Pt 4)**:911-919.

29. Hanke T, McMichael AJ, Dennis MJ, Sharpe SA, Powell LA, McLoughlin L*, et al.* Biodistribution and persistence of an MVA-vectored candidate HIV vaccine in SIV-infected rhesus macaques and SCID mice. *Vaccine* 2005; **23(12)**:1507-1514.

30. Hanke T, McMichael AJ, Samuel RV, Powell LA, McLoughlin L, Crome SJ*, et al.* Lack of toxicity and persistence in the mouse associated with administration of candidate DNA- and modified vaccinia virus Ankara (MVA)-based HIV vaccines for Kenya. *Vaccine* 2002; **21(1-2)**:108-114.

31. Stittelaar KJ, Kuiken T, de Swart RL, van AG, Vos HW, Niesters HG*, et al.* Safety of modified vaccinia virus Ankara (MVA) in immune-suppressed macaques. *Vaccine* 2001; **19(27)**:3700-3709.

32. Gomez CE, Najera JL, Jimenez EP, Jimenez V, Wagner R, Graf M*, et al.* Head-to-head comparison on the immunogenicity of two HIV/AIDS vaccine candidates based on the attenuated poxvirus strains MVA and NYVAC co-expressing in a single locus the HIV-1BX08 gp120 and HIV-1(IIIB) Gag-Pol-Nef proteins of clade B. *Vaccine* 2007; **25(15)**:2863-2885.

33. Mooij P, Balla-Jhagjhoorsingh SS, Koopman G, Beenhakker N, van HP, Baak I*, et al.* Differential CD4+ versus CD8+ T-cell responses elicited by different poxvirus-based human immunodeficiency virus type 1 vaccine candidates provide comparable efficacies in primates. *J Virol* 2008; **82(6)**:2975-2988.

34. Hirsch VM, Fuerst TR, Sutter G, Carroll MW, Yang LC, Goldstein S*, et al.* Patterns of viral replication correlate with outcome in simian immunodeficiency virus (SIV)-infected macaques: effect of prior immunization with a trivalent SIV vaccine in modified vaccinia virus Ankara. *J Virol* 1996; **70(6)**:3741-3752.

35. Seth A, Ourmanov I, Kuroda MJ, Schmitz JE, Carroll MW, Wyatt LS*, et al.* Recombinant modified vaccinia virus Ankara-simian immunodeficiency virus gag pol elicits cytotoxic T lymphocytes in rhesus monkeys detected by a major histocompatibility complex class I/peptide tetramer. *Proc Natl Acad Sci U S A* 1998; **95(17)**:10112-10116.

36. Seth A, Ourmanov I, Schmitz JE, Kuroda MJ, Lifton MA, Nickerson CE*, et al.* Immunization with a modified vaccinia virus expressing simian immunodeficiency virus (SIV) Gag-Pol primes for an anamnestic Gag-specific cytotoxic T-lymphocyte response and is associated with reduction of viremia after SIV challenge. *J Virol* 2000; **74(6)**:2502-2509.

37. Ourmanov I, Bilska M, Hirsch VM, Montefiori DC. Recombinant modified vaccinia virus ankara expressing the surface gp120 of simian immunodeficiency virus (SIV) primes for a rapid neutralizing antibody response to SIV infection in macaques. *J Virol* 2000; **74(6)**:2960-2965.

38. Ourmanov I, Brown CR, Moss B, Carroll M, Wyatt L, Pletneva L*, et al.* Comparative efficacy of recombinant modified vaccinia virus Ankara expressing simian immunodeficiency virus (SIV) Gag-Pol and/or Env in macaques challenged with pathogenic SIV. *J Virol* 2000; **74(6)**:2740-2751.

39. Garcia F, Bernaldo de Quiros JC, Gomez CE, Perdiguero B, Najera JL, Jimenez V*, et al.* Safety and immunogenicity of a modified pox vector-based HIV/AIDS vaccine candidate expressing Env, Gag, Pol and Nef proteins of HIV-1 subtype B (MVA-B) in healthy HIV-1-uninfected volunteers: A phase I clinical trial (RISVAC02). *Vaccine* 2011; **29(46)**:8309-8316.

40. Gomez CE, Najera JL, Perdiguero B, Garcia-Arriaza J, Sorzano CO, Jimenez V*, et al.* The HIV/AIDS Vaccine Candidate MVA-B Administered as a Single Immunogen in Humans Triggers Robust, Polyfunctional, and Selective Effector Memory T Cell Responses to HIV-1 Antigens. *J Virol* 2011; **85(21)**:11468-11478.

41. Guerra S, Gonzalez JM, Climent N, Reyburn H, Lopez-Fernandez LA, Najera JL*, et al.* Selective induction of host genes by MVA-B, a candidate vaccine against HIV/AIDS. *J Virol* 2010; **84(16)**:8141-8152.

# 
